# Linking anthocyanin diversity, hue, and genetics in purple corn

**DOI:** 10.1093/g3journal/jkaa062

**Published:** 2021-01-11

**Authors:** Laura A Chatham, John A Juvik

**Affiliations:** Department of Crop Sciences, University of Illinois at Urbana Champaign, Champaign, IL 61801, USA; Department of Crop Sciences, University of Illinois at Urbana Champaign, Champaign, IL 61801, USA

**Keywords:** anthocyanin, flavonoid biosynthesis pathway, maize pericarp, purple corn, genotyping-by-sequencing

## Abstract

While maize with anthocyanin-rich pericarp (purple corn) is rising in popularity as a source of natural colorant for foods and beverages, information on color range and stability—factors associated with anthocyanin decorations and compositional profiles—is currently limited. Furthermore, to maximize the scalability and meet growing demands, both anthocyanin concentrations and agronomic performance must improve in purple corn varieties. Using the natural anthocyanin diversity present in a purple corn landrace, Apache Red, we generated a population with variable flavonoid profiles—flavanol–anthocyanin condensed forms (0–83%), acylated anthocyanins (2–72%), pelargonidin-derived anthocyanins (5–99%), C-glycosyl flavone co-pigments up to 1904 µg/g, and with anthocyanin content up to 1598 µg/g. Each aspect of the flavonoid profiles was found to play a role in either the resulting extract hue or intensity. With genotyping-by-sequencing of this population, we mapped aspects of the flavonoid profile. Major quantitative trait loci (QTLs) for anthocyanin type were found near loci previously identified only in aleurone-pigmented maize varieties [*Purple aleurone1* (*Pr1*) and *Anthocyanin acyltransferase1* (*Aat1*)]. A QTL near *P1* (*Pericarp color1*) was found for both flavone content and flavanol–anthocyanin condensed forms. A significant QTL associated with peonidin-derived anthocyanins near a candidate S-adenosylmethionine-dependent methyltransferase was also identified, warranting further investigation. Mapping total anthocyanin content produced signals near *Aat1*, the aleurone-associated bHLH *R1 (Colored1)*, the plant color-associated MYB, *Pl1 (Purple plant1)*, the aleurone-associated recessive intensifier, *In1 (Intensifier1)*, and several previously unidentified candidates. This population represents one of the most anthocyanin diverse pericarp-pigmented maize varieties characterized to date. Moreover, the candidates identified here will serve as branching points for future research studying the genetic and molecular processes determining anthocyanin profile in pericarp.

## Introduction

With continued trends in consumer preference for less processed foods, rising demand for naturally pigmented food and beverages necessitates the development of economical natural colorant sources capable of matching the range of possible hues and stability of artificial colorants. Purple corn is a good source of anthocyanins, the water-soluble, red to purple pigments found in many fruits and vegetables. As a scalable commodity, corn could provide a natural colorant source capable of meeting growing consumer demands. However, wide-scale economic production will require breeding lines with both (1) appropriate hue and stability and (2) maximal anthocyanin production, all while maintaining yield and other agronomic traits. Accomplishing these goals will require identifying lines with desirable anthocyanin profiles and maximal anthocyanin production to backcross into current elite inbreds. With each round of backcrossing, selections must be made to minimize linkage drag while avoiding the loss of any factors associated with anthocyanin production. Anthocyanin biosynthesis requires a host of different structural genes and regulatory mechanisms, all of which have the potential to become unfixed upon backcrossing with nonpigmented lines. Loss of any of these factors could influence anthocyanin yield to varying degrees, illustrating the benefit of developing genomic resources to assist in breeding.

Kernel anthocyanin content has long been studied in maize due to its easily observable phenotype, but most research to date has focused on kernels with pigmented aleurone, the outermost, single-cell layer of the triploid endosperm. However, most varieties of interest for use as food and beverage colorant [often referred to as purple corn (Chatham *et al.* 2019b)], contain anthocyanin in the pericarp, the maternal, outermost layer of the kernel. Compared to aleurone, pericarp anthocyanin-pigmented lines comprise a relatively small portion of the available germplasm and thus have limited variability. Despite this disadvantage, lines with anthocyanin-pigmented pericarp often have concentrations an order of magnitude greater than aleurone lines (Paulsmeyer *et al.* 2017) and may offer more efficient extraction (Li *et al.* 2017). Moreover, established milling processes separating pericarp could provide valuable pigment-rich pericarp fractions and nonpigmented grain and germ fractions to be used for the production of food, feed, or fuel, creating a value-added product and more economical color source (Somavat *et al.* 2018). Furthermore, pigments extracted from maize kernels, a commonly consumed vegetable, are exempt from certification and can be labeled as “fruit or vegetable use for color” on food and beverage products in both the United States and EU [Code of Federal Regulations 2011; EFSA Panel on Food Additives and Nutrient Sources added to Food (ANS) 2013]. Other maize parts such as husk leaves and cob, which also offer a concentrated and easily extractable source of anthocyanins (de Pascual-Teresa *et al.* 2002; Li *et al.* 2008), face regulatory hurdles and require certification prior to use, making them an undesirable choice in the food and beverage industry.

Anthocyanins in purple corn consist primarily of glucosides and malonyl/dimalonyl glucosides of the anthocyanidins cyanidin, pelargonidin, and peonidin. Color is largely influenced by anthocyanidin type, shifting from orange/red to pink/red as the number of hydroxyl and methoxy groups on the B ring increases (Ananga *et al.* 2013). However, anthocyanidin type alone cannot account for the diversity in colors observed. Color stability and extract shelf life have been associated with the proportion of acylated anthocyanins (Zhao *et al.* 2017). Similarly, condensed forms, heterodimers consisting of a flavan-3-ol covalently linked to an anthocyanin, have been associated with altered hues and stability (Luna-Vital *et al.* 2017; Haggard *et al.* 2018) but have only been found in significant quantities in pericarp-pigmented varieties (Paulsmeyer *et al.* 2017).

**Figure 1:**
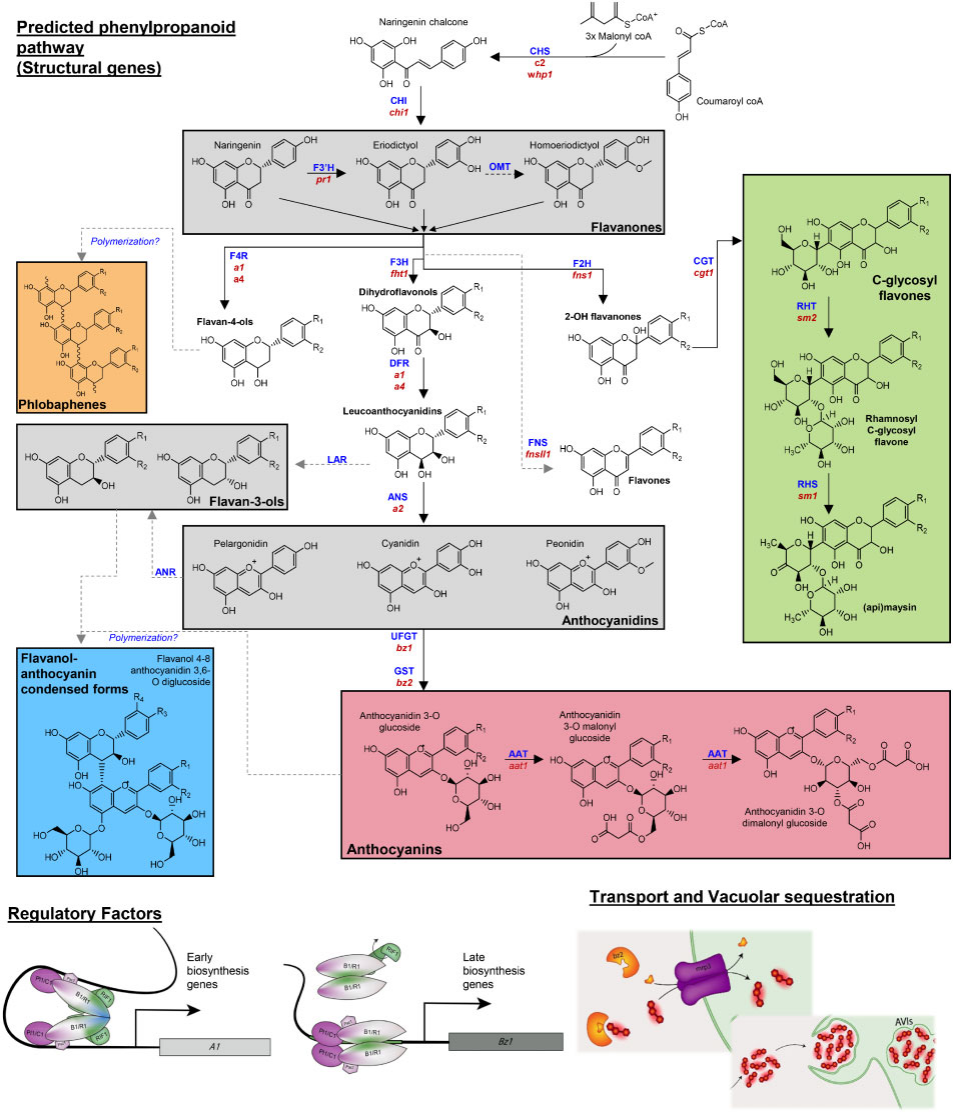
Expected flavonoid biosynthesis pathway in maize. Gray boxes indicate families of compounds that are intermediates in the pathway. Colored boxes represent endpoints of the pathway. Gene product abbreviations (blue) and genes (red) can be found in Table 1. Schematics at the bottom represent the predicted regulatory factors for early or late biosynthetic genes (left) and the trafficking and storage of anthocyanins in vacuoles (right).

Genetic factors controlling anthocyanidin type (*e.g.*, pelargonidin, cyanidin, peonidin) and anthocyanin decorations such as acylation and condensation with flavanols are of interest in purple corn because of their potential to influence the color and stability of extracts. The anthocyanin biosynthesis pathway in maize is well studied, and most structural genes (encoding nonregulatory products), with the exception of those controlling the formation of peonidin-based anthocyanins and flavanol-anthocyanin condensed forms, have been characterized (Figure 1, Table 1) (Cone 2007; Sharma *et al.* 2011). However, these structural genes were primarily identified in aleurone-pigmented varieties, and while the pathway is well conserved among plants and most structural genes are assumed to operate similarly in aleurone and pericarp, empirical evidence of this is lacking. *Pr1* (*Red aleurone1*) is known to control the ratio of cyanidin and pelargonidin in aleurone (Sharma *et al.* 2011), and more recently, an anthocyanin acyltransferase (*Aat1*) was identified in an aleurone-pigmented population with reduced proportions of acylated anthocyanins in some individuals (Paulsmeyer *et al.* 2018).

**Table 1:** Anthocyanin structural genes corresponding to abbreviated genes and gene products labeled in **Figure 1**

		Gene product	Gene model	Chromosome	Mb
***Structural***					
*** c2***	Colorless2	CHS (chalcone synthase)	Zm00001d052673	Chr 4	196.9
*** whp1***	White pollen1		Zm00001d007403	Chr 2	231.0
*** chi1***	Chalcone flavanone isomerase1	CHI (chalcone isomerase)	Zm00001d034635	Chr 1	298.6
*** chi3***	Chalcone flavanone isomerase3		Zm00001d012972	Chr 5	2.6
*** chi4***	Chalcone flavanone isomerase4		Zm00001d018278	Chr 5	217.8
*** chi5***	Chalcone flavanone isomerase5		Zm00001d016144	Chr 5	146.5
*** pr1***	Purple aleurone1	F3’H (flavonoid 3'-hydroxylase)	Zm00001d017077	Chr 5	184.5
*** Fht1***	Flavanone 3-hydroxylase1	F3H (flavonone 3-hydroxylase)	Zm00001d001960	Chr 2	3.6
*** Fht2***	Flavanone 3-hydroxylase2		Zm00001d029218	Chr 1	62.7
*** A1***	Anthocyaninless1	DFR/F4R (bifunctional dihydroflavonol 4-reductase/flavanone 4-reductase)	Zm00001d044122	Chr 3	219.9
*** A4***	Anthocyaninless4		Zm00001d011438	Chr 8	150.5
*** A2***	Anthocyaninless2	ANS (anthocyanidin synthase/leucoanthocyanidin dioxygenase)	Zm00001d014914	Chr 5	68.0
*** Fns1***	Flavone synthase1	F2H (flavonone 2-hydroxylase)	Zm00001d047452	Chr 9	130.7
*** Fnsii1***	Flavone synthase typeII1	FNS (flavone synthase)	Zm00001d024946	Chr 10	96.3
			Zm00001d024943	Chr 10	96.1
*** Cgt1***	C-glucosyl transferase1	CGT (c-glucosyl transferase)	Zm00001d037382	Chr 6	123.8
*** Sm1***	Salmon silk1	RHS (rhamnose synthase)	Zm00001d037784	Chr 6	137.7
*** Sm2***	Salmon silk2	RHT (rhamnosyl transferase)	Zm00001d006446	Chr 2	208.5
*** Bz1***	Bronze1	UFGT (UDP-glucose flavonol glycosyltransferase)	Zm00001d045055	Chr 9	11.2
*** Ufgt2***	UDP-flavonol-glycosyltransferase2		Zm00001d019256	Chr 7	24.2
*** Aat1***	Anthocyanin acyltransferase1	AAT (anthocyanin acyltransferase)	Zm00001d034925	Chr 1	305.7
***Regulatory***					
*** pl1***	Purple plant1	R2R3-MYB	Zm00001d037118	Chr 6	113.0
*** c1***	Colored aleurone1		Zm00001d044975	Chr 9	9.0
*** b1***	Colored plant1	bHLH (basic helix-loop-helix)	Zm00001d000236	Chr 2	19.0
*** r1***	Colored1		Zm00001d026147	Chr 10	139.8
*** hopi1***	Hopi r1/b1 family member1		Zm00001d026147		
*** lc1***	Red leaf color1		Zm00001d026147		
*** sn1***	Scutellar node color1		Zm00001d026147		
*** pac1***	Pale aleurone color1	WDR (WD repeat protein)	Zm00001d017617	Chr 5	201.8
*** a3***	Anthocyaninless3	Unknown	no gene model		
*** p1***	Pericarp color1	MYB	Zm00001d028842	Chr 1	48.4
*** in1***	Intensifier1	bHLH-like inhibitor	Zm00001d019170	Chr 7	20.1
*** rif1***	R-interacting factor1	EMSY-like/ENT domain containing protein	Zm00001d019922	Chr 7	76.6
***Transport/Storage***					
*** bz2***	Bronze2	GST (glutathione *S*-transferase)	Zm00001d032969	Chr 1	245.0
*** mrp3***	Multidrug resistance-associated protein3	MRP (multidrug resistance-associated protein, ABC transporter)	Zm00001d045269	Chr 9	17.5

While structural genes are the primary focus for optimizing stability and hue, regulatory factors are the primary target for maximizing overall anthocyanin concentrations. The major regulatory players have been identified, but current models in maize are simplistic compared to those in other anthocyanin-producing species (Chatham *et al.* 2019b). The canonical anthocyanin regulatory complex consists of an R2R3-MYB protein, a bHLH (basic helix-loop-helix) protein, and a WDR (WD-repeat) domain containing protein, collectively referred to as the MBW complex (Lloyd *et al.* 2017). Typically, *Colored aleurone1* (*C1*, MYB), *Colored1* (*R1*, bHLH), and *Pale aleurone color1* (*Pac1*, WDR) form the regulatory complex and activate structural genes in aleurone tissue. A bHLH-like recessive intensifier *in1* (*intensifier1*) with similarity to *R1* increases anthocyanin pigmentation in aleurone (Burr *et al.* 1996), and more recently, an EMSY-like factor, *RIF1* (*R interacting factor1*), was found to act as a switch to help control activation of structural genes (Hernandez *et al.* 2007). An analogous MBW complex likely forms to regulate anthocyanin biosynthesis in plant and pericarp tissue. *Booster1*(*B1*) and *Plant color1* (*Pl1*) are the bHLH and MYB regulatory factors, respectively, most often associated with regulation in plant tissues. However, certain alleles of *R1* (*e.g.*, *R1-ch)* operate in pericarp and certain *B1* alleles (*e.g.*, *B1-Peru*) operate in aleurone (Portwood *et al.* 2019). Whether *Pac1, RIF1*, or *IN1* function in pericarp has yet to be determined, and no pericarp-specific alleles or homologs have been identified. Furthermore, R2R3-MYB repressors of anthocyanin biosynthesis have been identified in a number of other species, yet none have been found in maize (Albert *et al.* 2014). MYB repressors associated with the phenylpropanoid pathway and downstream lignin biosynthesis have been identified (Agarwal *et al.* 2016).

In addition to structural and regulatory factors, factors associated with cellular transport and storage of anthocyanins could also play a key role in anthocyanin content. In maize, *Bz2* (*Bronze2*) encodes a glutathione S-transferase which assists in anthocyanin transport and is required to prevent anthocyanin oxidation, resulting in a bronze-colored kernel (Marrs *et al.* 1995). *Mrp3* (*Multidrug resistance-associated protein3*) produces an ABC transporter that shuttles anthocyanins into the vacuole (Goodman *et al.* 2004). Both were identified and studied in aleurone-pigmented lines. In other anthocyanin-producing species, two other mechanisms of vacuolar localization have been identified in addition to the GST/ABC transporter system—MATE transporters and vesicle trafficking. MATE transporters, either as H^+^/flavonoid antiporters or in combination with other vacuolar proton gradient maintaining mechanisms, have been identified in *Arabidopsis, Medicago*, tomato *(S. lycopersicum*), and radish (*R. sativa*) (Debeaujon *et al.* 2001; Mathews *et al.* 2003; Gomez *et al.* 2009; Zhao *et al.* 2011; Kitamura *et al.* 2016; M’mbone *et al.* 2018). Support for vesicle trafficking of anthocyanins and a subsequent autophagy-like mechanism of vacuolar transport has been found in *Arabidopsis, Brassica napus*, and grapevine (*V. vinifera)*. Some evidence for this mechanism exists in maize. Anthocyanin-containing vesicles and anthocyanin vacuolar inclusions (AVIs) were shown to fuse in response to light (Chanoca *et al.* 2015).

In addition to anthocyanins, several other flavonoids are present in maize. Phlobaphenes, orange to brick red pigments accumulating in cob and pericarp, are polymers of the 3-deoxyflavonoids, luteoforol or apiforol, and share the early steps of their biosynthesis and thus carbon flux with anthocyanins. However, phlobaphenes are insoluble in water and thus are expected to have little or no effect on anthocyanin extracts when both are present in the same ear. The structural genes responsible for phlobaphene biosynthesis are controlled by *Pericarp color1* (*P1*), an R2R3 MYB similar to *C1*/*Pl1* but functioning without a bHLH partner. *P1* also regulates C-glycosyl flavone biosynthesis, a group of compounds that includes maysin, an insecticidal compound conferring resistance to corn earworm (*Helicoverpa zea*) (Grotewold *et al.* 1994; Byrne *et al.* 1996). Recently, C-glycosyl flavone-rich maize extract added to maize anthocyanin-rich extract was shown to significantly alter extract hue and stability (Chatham *et al.* 2019a). While C-glycosyl flavone content and function in silks is well studied for its insecticidal properties (Byrne *et al.* 1996), flavone and anthocyanin co-occurrence in pericarp of the kernels and its effect on extract hue have not been studied or reported previously.

With multiple endpoints, branch points, and shared precursors, flux through the flavonoid pathway in maize is complex. Breeding for maximal anthocyanin content is likely to benefit greatly from a better understanding of the mechanisms regulating this flux. Similarly, identifying factors associated with extract hue and stability can help prevent their loss during the process of backcrossing and maximizing color content. Here, we seek to understand how various anthocyanin profiles affect extract hue and color intensity in a diverse purple corn landrace, linking anthocyanin chemistries to the resulting extract phenotype. We then link phenotype to genotype, identifying genetic loci underlying anthocyanin diversity and color in purple corn.

## Materials and methods

### Plant materials

Seeds of a purple corn landrace, Apache Red (AR), were purchased from Siskiyou Seeds (Williams, OR) (S_0_ population) and grown out to produce S_1_ seeds. S_1_ seeds were analyzed for anthocyanin content using HPLC and seven anthocyanin-rich lines were advanced while progeny with little or no anthocyanin present in the pericarp were discarded. Selected anthocyanin-rich S_1_ ears along with a sample of S_0_ seeds from the original AR source were grown and self-pollinated, producing 181 S_2_s and 8 new S_1_s, which were also analyzed using HPLC. The available S_2_ lines were genotyped as described below. A selection (9 lines) of highly pigmented S_2_ seeds was grown in a winter nursery to produce 86 S_3_s. The following season, a selection of anthocyanin containing S_1_, S_2_, and S_3_ lines spanning the available anthocyanin diversity in our AR lines was planted and S_2_, S_3_, and S_4_ seeds were recovered. Of these, anthocyanin-rich lines were selected from each S_1_, S_2_, and S_3_ parent for genotyping and phenotyping (1148 lines, 103 S_2_s, 730 S_3_s, and 315 S_4_s). To this, 69 samples containing no or minimal anthocyanin content and representing the original 7 S_1_ families were added and genotyped and phenotyped to supplement the dataset. A schematic showing population construction can be found in Supplementary Figure S1.

### Extractions

The anthocyanin extraction method was based on previously published protocols and findings regarding the efficiency of anthocyanin extraction protocols (Paulsmeyer *et al.* 2017; Chatham *et al.* 2018). Briefly, a subset of kernels from each line was ground to a fine powder using a coffee grinder and 1 g of powder was weighed out for extraction. Samples were extracted in 5 ml of aqueous 2% formic acid for 1 h at 50°C with constant agitation. Extracts were centrifuged and filtered through a 25-mm, 0.45-µm Millex LCR PTFE syringe filter (Millipore, Billerica, MA) prior to HPLC analysis and optical measurement with UV-visible spectroscopy.

### HPLC analysis

Anthocyanin and flavone content was measured using an Agilent 1100 series high-performance liquid chromatography (HPLC) system and diode array detector (DAD) (Santa Clara, CA) in a similar manner to previous reports (Chatham *et al.* 2018). The stationary phase consisted of a Poroshell 120 SB-C18 100 mm × 4.6 mm, 2.7 µm column (Agilent), and the mobile phase was a two-solvent mixture, with 2% formic acid in water as solvent A, and acetonitrile as solvent B. The protocol began at 93% solvent A and 7% solvent B, and solvent B increased linearly to 18% over 30 min. Column temperature was kept at 30°C, an injection volume of 20 µl was used, and absorbance was measured at 520 for anthocyanin content and 340 nm for flavone content using the DAD. ChemStation (rev A10.02) software (Agilent) was used for integration and anthocyanins were quantified using standard curves of cyanidin, pelargonidin, and peonidin 3-glucosides, while flavones were quantified using *C-*hexosyl apigenin compounds (vitexin and isovitexin) (Extrasynthese, Genay, France). Identities were confirmed by mass spectrometry as described previously (Chatham *et al.* 2018). To ensure consistency between batches of samples and retention times, an AR mixture was created by combining ground powder from multiple AR lines, a sample of which was extracted and run on HPLC with each batch of samples. This was done to ensure consistency in compound retention times and peak areas across batched extractions and HPLC runs.

### UV-Visible spectroscopy

Absorbance was recorded between 380 and 780 nm on a Synergy 2 multi-well plate reader (BioTek, Winooski, VT, USA) using a 96-well plate. Each extract was run in triplicate, and the maximum absorbance (Abs_max_) and the wavelength at Abs_max_ (λ_max_) were recorded.

### Statistical analysis

Anthocyanin content, flavone content, and all other factors used in analyzing the relationship between various anthocyanin species and hue were normalized before plotting, and all statistics were calculated using R.

### GBS library construction and sequencing

Fourteen S_2_, S_3_, and S_4_ seeds of each line (1148 lines in total) were sown in 11 × 21 cell propagation trays (Proptek, Watsonville, CA) filled with a soilless seedling mix (Sunshine Redi-Earth Plug & Seedling; SunGro, Agawam, MA) and grown in a greenhouse at the University of Illinois Plant Care Facility. Once seedlings had emerged, tissue of a similar size and proportion was harvested from at least seven seedlings of each line, depending on germination rate. Samples were frozen to −20°C, lyophilized, and ground in 15-ml conical tubes with stainless steel ball bearings using an automated tissue homogenizer (SPEX SamplePrep, Metuchen, NJ). A 96-well optimized CTAB DNA extraction protocol was used for DNA isolation, and concentrations were determined using the Quant-iT PicoGreen dsDNA Assay Kit (Thermo Fisher Scientific, Waltham, MA). GBS libraries were constructed as described previously using a double restriction enzyme digest with *HinP1I* and *PstI* (Elshire *et al.* 2011; Poland *et al.* 2012). All lines were multiplexed into three lanes and sequenced using the Illumina HiSeq 4000 system with single-end 100 nucleotide reads at the Roy J. Carver Biotechnology Center at the University of Illinois in Urbana, IL.

### SNP discovery and association mapping

SNP discovery was carried out using the TASSEL 5.0 GBS v2 pipeline (Bradbury *et al.* 2007). Reads were aligned to the *Zea Mays* B73 reference genome version 4 (Jiao *et al.* 2017) using Bowtie 2 (Langmead and Salzberg 2012), and minor allele frequency was set to 0.05. This generated 149,342 SNP sites with an average depth of 12 and average proportion covered of 0.65. With imputation, performed using Beagle 5.0 (Browning *et al.* 2018), and filtering, a total of 41,986 nonredundant SNPs per sample were generated. Association mapping was performed using GAPIT (Genome Association and Prediction Integrated Tool) in R (Lipka *et al.* 2012) for anthocyanin and flavone compounds, absorbance, and extract λ_max_. Variables were transformed as needed prior to mapping to achieve a more normal data spread using either a log (Abs_max_, total flavone content), square root (total anthocyanin content), or rank-based inverse normal transformation (condensed forms). GWAS was also run individually for each chromosome using a leave-one-out method of calculating kinship to retain maximal power for detecting significant associations (Chen and Lipka 2016). Seven principle components were used to adequately account for population structure in addition to the kinship matrix provided by GAPIT.

### Methyltransferase candidates

The amino acid sequence of *VvAOMT1*, an S-adenosyl-L-methionine-dependent methyltransferase, with 3-*O-*methyltransferase activity (Hugueney *et al.* 2009) was blasted against the maize genome to identify homologous candidates in maize. These candidates along with known caffeoyl-co A *O*-methyltransferases (CcoAOMTs), anthocyanin *O-*methyltransferases (AOMTs), and caffeic acid *O-*methyltransferases (CaOMTs) gathered from NCBI. Candidate sequence selection was based in part on phylograms from Provenzano *et al.* (2014) and Du *et al.* (2015). Sequences were aligned in R using the DECIPHER (Wright 2016) package, and a phylogram was created using the phangorn package (Schliep 2011). Alignment of leucoanthocyanidin reductases and anthocyanidin reductase was performed in a similar manner using the same R packages.

### Data availability

Lines from the Apache Red population are available by request. All genotype and phenotype datasets have been made available via Figshare under project name “Linking anthocyanin diversity, hue, and genetics in purple corn”. https://doi.org/10.6084/m9.figshare.11991552 and https://doi.org/10.6084/m9.figshare.11991555.

Supplementary material is available at *G3* online.

## Results

### Anthocyanin diversity

**Figure 2:**
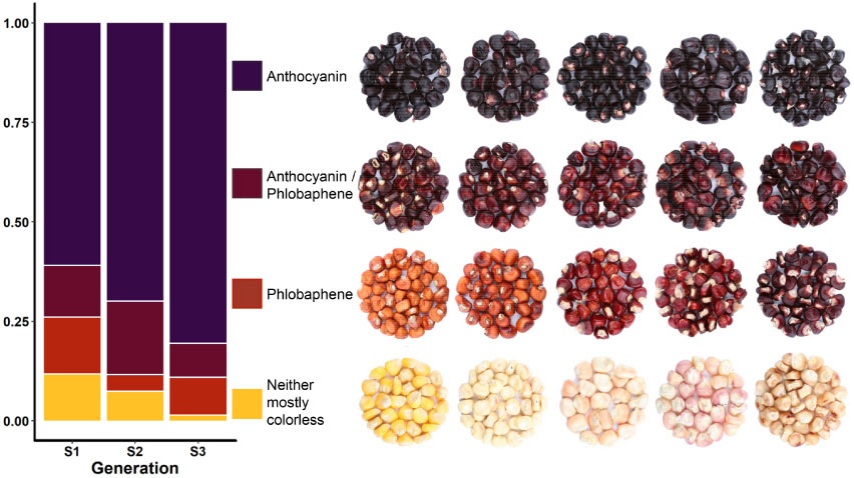
Proportions of lines from each generation containing anthocyanin and phlobaphenes based on visual assessment. Some dark kernels may have phlobaphenes present that were not visually undetectable; likewise, phlobaphene rich lines may have had undetectable amounts of anthocyanin. Kernel pictures show ranges for each category. Generations are labeled based on maternal pericarp (*i.e.*, S2, S3, and S4 plants have maternal pericarp representative of the S1, S2, and S3 generations, respectively).

Anthocyanin content was measured at each stage of population development used to calculate heritability based on parent offspring correlations (Foolad *et al.* 2002). Anthocyanin content heritability ranged from 0.44 to 0.57, suggesting improvement can be made through selection. Figure 2 shows a summary of kernel colors obtained from the Apache Red (AR) population. AR S_0_ seeds were all dark purple, indicative of anthocyanins in pericarp, but S_1_s varied in color. Most were dark purple, anthocyanin-containing, but we observed a range of water-insoluble phlobaphene-containing kernels colored from orange to purple-red and some kernels that were colorless or had minimal anthocyanin or phlobaphene pigmentation.

The wavelength at maximum absorption (λ_Max_) of anthocyanin lines ranged from 497 nm to 521 nm. Anthocyanin and C-glycosyl flavones were highly concentrated compared to previous reports (1598 µg/g whole corn and 1904 µg/g, respectively, Table 2) (Paulsmeyer *et al.* 2017; Chatham *et al.* 2018). Many lines were abundant in both; that maximal flavone: anthocyanin ratio was 11:1.

**Table 2:** Diversity in anthocyanin content of 1152 Apache Red lines

Category	Range
Acylated anthocyanins	2–72%
Malonylated	0–43%
Dimalonylated	0–57%
Pelargonidin-derived anthocyanins	5–99%
Cyanidin-derived anthocyanins	0–86%
Peonidin-derived anthocyanins	0–50%
Flavanol–anthocyanin condensed forms	0–83%
Maximum total flavone content	1904 µg/g
Maximum total anthocyanin content	1598 µg/g

**Figure 3:**
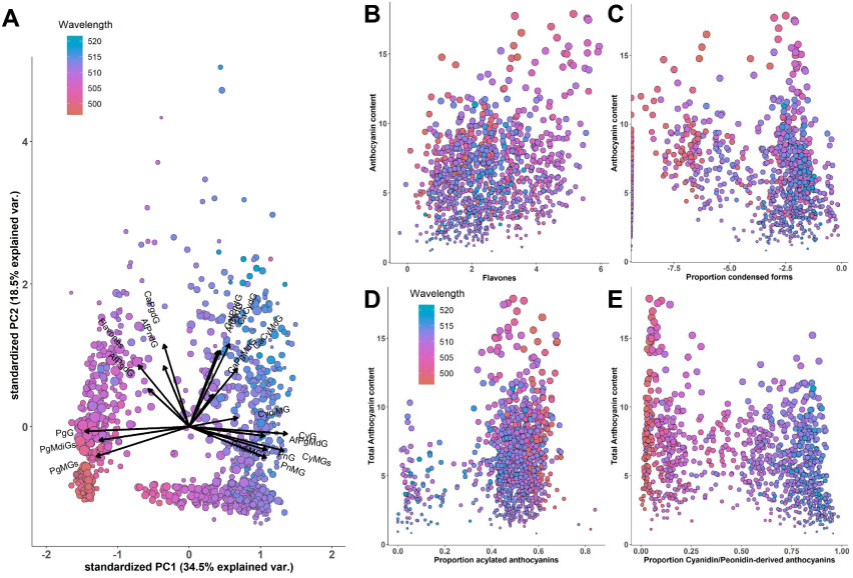
(A) PCA biplot using proportions of total HPLC peak areas and total flavone content. Effects of flavone content (B, log transformed, μg/ml) and proportions of condensed (C, log transformed), acylated (D), and cyanidin-/peonidin-derived (E) anthocyanins on normalized total anthocyanin content. Points are sized according to normalized total anthocyanin content (square root transformed, μg/ml). Observations are colored by wavelength (λ_Max_), and size corresponds to total anthocyanin content.

Figure 3A illustrates the overall chemical diversity in the AR population using principal component analysis (PCA) based on proportions of total anthocyanin content attributed to each anthocyanin species. An HPLC chromatogram with peaks used for this analysis can be found in Supplementary Figure S2 and PCA loadings and peak identities in Table 3. Each anthocyanin was confirmed using retention time and previous mass spectrometry results (Chatham *et al.* 2018).

**Table 3:** Summary of variables used for PCA and loadings for PC1 and PC2

Peak	Anthocyanin		PC1	PC2
9	CyG	Cyanidin 3-glucoside	0.35	
13	CyMGs	Cyanidin malonylglucosides	0.338	−0.117
12	PnG	Peonidin 3-glucoside	0.277	−0.113
15	PnMG	Peonidin 3-malonylglucoside	0.275	−0.152
11	AfPgMdG	Afzelechin-(4,8)-cyanidin-3-malonylglucoside-5-glucoside	0.269	
5	CaCyMdG	Catechin-(4,8)-cyanidin-3-malonylglucoside-5-glucoside	0.172	0.284
16	CydiMG	Cyanidin 3-5-dimalonylglucoside	0.172	
1	CaCydG	Catechin-(4,8)-cyanidin-3,5-diglucoside	0.144	0.404
4	AfCydG	Afzelechin-(4,8)-cyanidin-3,5-diglucoside	0.111	0.372
3	CatPndG	Catechin-(4,8)-peonidin-3,5-diglucoside	0.102	0.37
7	AfPgdG	Afzelechin-(4,8)-pelargonidin-3,5-diglucoside	−0.146	0.185
	Flavone	Sum of di-*C, C*-hexosyl apigenin, *C*-hexosyl-*C-*pentosyl apigenin, *C-*pentosyl-*C-*hexosyl apigenin, and *C-*hexosyl apigenin	−0.181	0.3
17	PgMdiGs	Pelargonidin dimalonylglucosides	−0.321	
14	PgMGs	Pelargonidin malonylglucosides	−0.331	−0.145
10	PgG	Pelargonidin 3-glucoside	−0.372	
2	CaPgdG	Catechin-(4,8)-pelargonidin-3,5-diglucoside		0.401
6	CaPgMdG	Catechin-(4,8)-pelargonidin-3-malonylglucoside-5-glucoside		0.158
8	AfPndG	Afzelechin-(4,8)-peonidin-3,5-diglucoside		0.297
18	PndiMG	Peonidin 3-5-dimalonylglucoside		

Peaks correspond to Supplementary Figure S2B.

The first principal component (PC1) explained 34.5% of the variability in the dataset and represents a contrast of pelargonidin *versus* cyanidin-derived anthocyanins. This is enforced by the color gradient across Figure 2A, in which point color represents wavelength. The λ_Max_ for pelargonidin 3-glucoside (PgG) is ∼496, the λ_Max_ for cyanidin 3-glucoside (CyG) is ∼510 (Giusti and Wrolstad 2001), and a gradient from low to high can be seen across the *x*-axis in the biplot. The second PC explained 18.5% of the variability and was related to the presence of condensed forms and flavones (high PC2 value) or acylated forms (low PC2 value). Wavelength also appeared to increase with PC2, suggesting condensed forms and flavones may play a role in hue.

### Relationship between anthocyanin profiles and λ_Max_

To further analyze the relationship between anthocyanin profiles and hue, λ_Max_ was modeled using PC1 and PC2. PC1, PC2, and their interaction were all significant (*P* < 0.05). We also looked at the major factors associated with PC1 and PC2 based on loadings for each component (Supplementary Figure S2, Table 3). These factors, including proportions of condensed, acylated, and cyanidin-/peonidin-derived forms (a measure of pelargonidin *versus* cyanidin) and flavone content, were plotted against λ_Max_ to identify correlations and create a complex global model from which the most parsimonious model was selected based on the lowest AIC (Akaike information criterion) score, an estimate of model quality. Collinearity between variables (as seen in PCA biplot in Figure 3) and the resulting multi-way interactions between factors make interpretation of the model and gauging whether these interactions have a biochemical basis difficult. Nonetheless, cyanidin content especially (*P* = 1.09e−235) and the other main effects [flavone, *P* = 7.66e−21; condensed, *P* = 9.65e−30; acylated (*P* = 3.67e−8)] were significant, suggesting each can be considered to influence λ_Max_ and overall color in AR lines.

### Relationship between anthocyanin profiles, total concentration, and Abs_max_

Flavone and anthocyanin contents were positively correlated (*r* = 0.28, *P* < 2.2e−16), but the proportion of anthocyanins present as flavanol–anthocyanin condensed forms was negatively correlated with total anthocyanin content (*r* = −0.27, *P* = 6.24e−16). Proportion of acylated anthocyanins was positively correlated with anthocyanin content (*r* = 0.23, *P* = 1.51e−14), and the proportion of cyanidin-derived anthocyanins was negatively associated with anthocyanin content (*r* = −0.31, *P* < 2.2e−16) (Figure 3). Both anthocyanin and flavone contents were highly correlated with Abs_max_ (*r* = 0.92, *P* = 2.2e−16; *r* = 0.43, *P* < 2.2e−16). In contrast, neither the proportion of condensed forms nor the proportion of acylated forms was significantly correlated with Abs_max_ (*P* = 0.15, *P* = 0.07). Flavonoid contents were also compared with maximal absorbance (Abs_max_) in the ∼495–525 nm range, a value indicative of color intensity and often used in a method for determining anthocyanin content (Supplementary Figure S2) (Giusti and Wrolstad 2001). Both anthocyanin and flavone contents were correlated with Abs_max_.

From a global model using all factors described above, the most parsimonious model was chosen. In this model, anthocyanin content, condensed forms, flavones, and the interactions between them were all significant, but anthocyanins and condensed forms had the smallest *P* values and largest effect sizes by far. Variables were scaled so that effect sizes can be directly compared. The anthocyanin term had a scaled effect size of 1.5 standard deviations from the mean and a *P*-value too low to estimate in R. The condensed form factor had a scaled effect size of 0.5 (*P* = 2.7e−77), and the interaction between anthocyanin and condensed forms had an effect size of 0.6 (*P* = 7.3e−59).

### Association mapping

**Figure 4:**
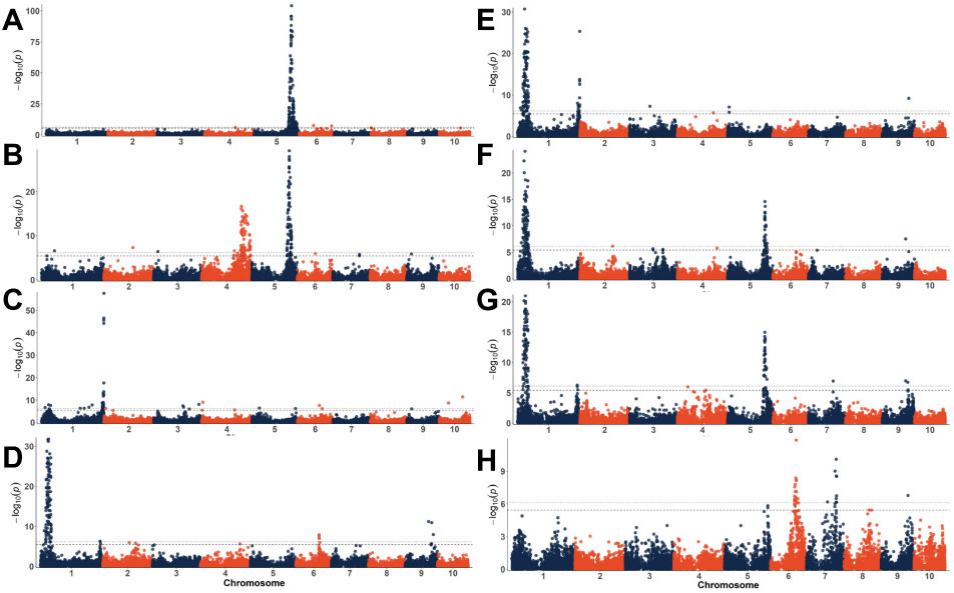
Manhattan plots for anthocyanin types, decorations, and flavone content. Percent cyanidin-derived anthocyanins (A), the percent peonidin of all cyanidin- and peonidin-derived anthocyanins (*i.e.*, peonidin/(peonidin + cyanidin)) (B), the proportion of acylated anthocyanins (peaks 13–18) relative to the total anthocyanin content (C), total condensed forms (peaks 1–8, 11) (D), the proportion of condensed forms relative to the total anthocyanin content (E), condensed forms measured as a proportion of molar flux (sum of flavone, anthocyanin, and condensed forms in each line in moles (F), total C-glycosyl flavones (G), and proportions of C-hexosyl-C-pentosyl apigenin (H). Dotted lines indicate the experiment-wide significance thresholds using the Bonferroni correction. (α_E_=0.05 and α_E_=0.01)

**Figure 5:**
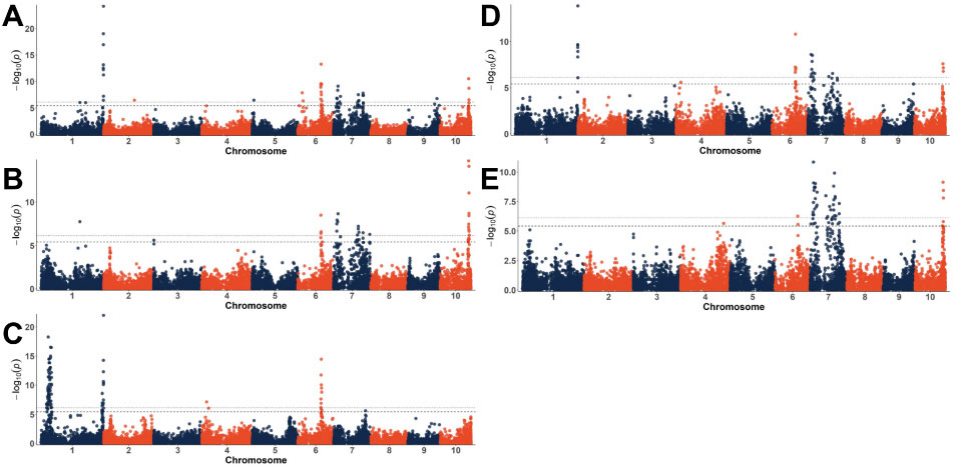
Manhattan plots for total anthocyanin content in μg/ml (A, B; sqrt transformed), the molar proportion of anthocyanins (C) and maximum absorbance (D, E; log transformed). A and D contain data from the full population while B and E contain only the lines with proportions of acylated anthocyanins between 0.35 and 0.7. Dotted lines indicate the experiment-wide significance thresholds using the Bonferroni correction. (α_E_=0.05 and α_E_=0.01).

Mapping was performed for each of the factors used in modeling λ_Max_, including cyanidin, pelargonidin, and peonidin content, anthocyanin decorations—acylation and condensation, and C-glycosyl flavone content (Figure 4). Mapping was also performed using both Abs_max_ and total anthocyanin content (Figure 5) for the entire AR population and a truncated version of the population. To map molar flux through the flavonoid pathway, anthocyanins, flavones, and condensed forms were converted to molar amounts and the proportion of the total molar amount was mapped. Significant SNPs were identified for each factor and are listed in Table 4.

**Table 4:** Significant SNPs^*^ for each mapped variable

	Variable	Range of significant SNPs				SNP with minimum *P*-value			
		*Chr*		*Min (Mb)*	*Max (Mb)*	*Position (Mb)*	*P-value*	*Effect*	*MAF*
Anthocyanidin type	*Proportion cyaniding*		4	152.22	152.22	152.22	1.54E−06	−0.09	0.15
		^**^	5	168.75	211.21	184.86	6.96E−105	0.31	0.48
		^**^	6	71.57	71.57	71.57	4.10E−08	−0.13	0.03
		^**^	6	161.00	161.00	161.00	6.09E−08	0.12	0.03
			8	0.82	0.82	0.82	2.94E−06	0.61	0.50
			10	104.61	104.61	104.61	3.51E−06	0.12	0.02
	*Proportion peonidin/(cyanidin + peonidin)*	^**^	1	64.04	64.04	64.04	2.72E−07	−0.15	0.17
		^**^	2	142.74	142.74	142.74	5.20E−08	−0.09	0.06
		^**^	3	20.69	20.69	20.69	4.00E−07	0.10	0.02
		^**^	4	157.82	240.70	195.50	2.13E−17	0.07	0.31
		^**^	5	172.18	196.29	184.02	5.01E−30	−0.08	0.50
			6	88.67	88.67	88.67	1.21E−06	0.07	0.04
			7	131.25	131.58	131.58	1.77E−06	−0.06	0.05
			9	23.85	23.85	23.85	1.35E−06	−0.15	0.03
Anthocyanin decorations—acylation	*Proportion of acylated anthocyanins*	^**^	1	17.95	45.12	35.49	1.28E−08	0.08	0.10
		^**^	1	190.18	190.18	190.18	4.92E−07	−0.09	0.03
		^**^	1	209.72	209.72	209.72	4.73E−07	−0.58	0.50
		^**^	1	233.55	233.55	233.55	1.59E−07	−0.11	0.03
		^**^	1	249.31	249.31	249.31	1.60E−08	0.14	0.10
		^**^	1	298.41	306.42	306.42	2.47E−58	−0.17	0.19
		^**^	2	8.13	8.13	8.13	5.96E−07	0.07	0.10
		^**^	3	143.43	174.37	143.43	3.98E−08	0.06	0.09
		^**^	3	221.63	221.63	221.63	8.73E−08	−0.08	0.07
		^**^	4	2.54	5.97	5.97	1.02E−09	−0.15	0.02
		^**^	4	160.96	160.96	160.96	2.60E−08	−0.08	0.04
		^**^	5	36.21	36.21	36.21	3.74E−07	−0.07	0.08
		^**^	5	213.51	213.51	213.51	6.84E−07	0.14	0.01
		^**^	6	107.49	121.60	107.49	2.65E−08	−0.11	0.03
			9	23.85	23.85	23.85	8.87E−07	0.17	0.03
		^**^	10	45.38	45.38	45.38	2.17E−09	−0.05	0.12
		^**^	10	115.12	115.12	115.12	4.13E−12	0.11	0.04
Anthocyanin decorations—flavanol–anthocyanin condensed forms	*Total condensed forms*	^**^	1	14.36	59.16	37.58	1.74E−31	−0.50	0.44
		^**^	1	294.06	295.91	294.13	5.80E−07	−0.29	0.06
			2	130.57	130.57	130.57	1.12E−06	0.43	0.05
			2	161.59	161.59	161.59	1.58E−06	0.33	0.07
			4	195.24	195.24	195.24	2.51E−06	−0.34	0.06
		^**^	6	113.77	117.74	113.98	1.25E−08	0.21	0.15
		^**^	9	115.42	138.71	115.42	6.33E−12	−0.47	0.32
	*Percent condensed forms*	^**^	1	14.36	54.03	35.49	1.80E−31		0.09
		^**^	1	295.59	300.43	295.59	1.00E−08		0.05
		^**^	1	302.39	306.42	306.42	4.54E−26		0.18
		^**^	3	100.32	100.32	100.32	5.46E−08		0.05
			4	176.64	174.64	176.64	2.14E−06		0.07
		^**^	5	7.62	7.70	7.62	8.55E−08		0.09
		^**^	9	130.35	130.35	130.35	6.64E−10		0.14
	*Condensed forms molar proportion*	^**^	1	19.59	54.25	37.58	8.22E−25	−1.28	0.44
		^**^	2	161.59	161.59	161.59	7.12E−07	1.06	0.07
			3	115.24	115.24	115.24	2.13E−06	0.66	0.07
			3	164.11	164.11	164.11	2.95E−06	−0.50	0.16
			4	195.24	195.24	195.24	1.70E−06	−1.10	0.06
		^**^	5	178.64	194.54	184.02	2.75E−15	0.66	0.50
		^**^	9	115.42	115.42	115.42	3.04E−08	−1.13	0.32
C-Glycosyl flavones	*Total flavone content*	^**^	1	27.09	53.46	38.98	1.01E−21	0.45	0.45
		^**^	1	294.06	296.60	294.06	5.36E−07	0.40	0.06
			4	52.19	52.19	52.19	1.06E−06	−0.48	0.05
			4	140.38	140.38	140.38	3.63E−06	−0.28	0.11
		^**^	5	175.54	193.62	183.40	1.07E−15	−0.46	0.44
		^**^	7	121.75	121.75	121.75	1.14E−07	−0.29	0.17
		^**^	9	115.42	127.22	115.42	1.08E−07	−0.47	0.32
	*Proportion C-hexosyl, C-pentosyl apigenin*		5	210.19	210.19	210.19	1.48E−06	0.04	0.20
		^**^	6	115.85	135.53	124.82	1.38E−12	−0.05	0.47
		^**^	7	102.41	102.41	102.41	6.73E−07	0.04	0.25
		^**^	7	138.23	146.65	144.83	7.91E−11	−0.07	0.10
			8	122.21	134.71	122.21	3.80E−06	0.05	0.08
		^**^	9	129.91	129.91	129.91	1.73E−07	0.06	0.11
Anthocyanin content	*Total anthocyanin content (sqrt)*		1	190.39	219.17	190.39	9.17E−07	−0.77	0.08
		^**^	1	305.34	306.42	306.42	5.78E−25	−1.54	0.19
		^**^	2	150.38	150.38	150.38	3.32E−07	−0.89	0.06
		^**^	5	8.56	8.56	8.56	3.40E−07	0.54	0.31
		^**^	6	7.25	25.97	21.26	1.38E−08	−1.39	0.07
		^**^	6	112.48	119.93	113.98	5.49E−14	1.21	0.15
		^**^	7	16.90	34.79	22.61	7.35E−10	0.65	0.48
		^**^	7	117.64	146.65	145.45	1.62E−08	0.60	0.10
		^**^	9	132.37	154.32	143.53	1.71E−07	1.13	0.07
		^**^	10	139.50	140.87	139.50	3.31E−11	1.32	0.27
	*Total anthocyanin content (sqrt, truncated dataset)*	^**^	1	190.39	190.39	190.39	1.89E−08	−0.93	0.08
			3	1.40	1.40	1.40	2.43E−06	−3.14	0.07
		^**^	6	112.79	113.98	113.00	3.25E−09	−0.69	0.17
		^**^	7	16.83	22.61	22.61	2.22E−09	0.62	0.48
			7	34.79	34.79	34.79	1.00E−06	0.82	0.10
		^**^	7	112.72	125.31	121.75	6.29E−08	−0.95	0.16
		^**^	7	177.72	177.72	177.72	5.43E−07	1.30	0.05
		^**^	10	137.27	142.86	139.50	1.73E−15	1.68	0.28
		^####^							
	*Molar proportion anthocyanins*	^**^	1	27.09	52.50	35.49	5.52E−19	1.07	0.10
		^**^	1	298.41	306.42	306.42	1.02E−22	−0.69	0.19
		^**^	4	23.41	33.19	23.41	7.35E−08	−0.95	0.05
		^**^	6	112.48	116.70	113.98	3.60E−15	0.76	0.15
			7	156.44	156.44	156.44	2.43E−06	0.55	0.08
Truncated dataset	*Abs_max_ (log)*	^**^	1	305.34	306.42	305.59	1.33E−14	−0.34	0.19
			4	23.41	23.41	23.41	2.61E−06	−0.38	0.05
		^**^	6	113.00	116.37	113.98	1.55E−11	0.32	0.15
		^**^	7	15.15	34.79	16.90	2.57E−09	0.05	0.17
		^**^	7	102.83	145.54	121.55	2.90E−07	0.23	0.14
		^**^	10	139.50	140.87	139.50	2.61E−08	0.32	0.27
	*Abs_max_ (log, truncated dataset)*		4	214.49	214.49	214.49	2.31E−06	−0.23	0.09
		^**^	6	112.89	113.98	113.00	5.59E−07	0.30	0.16
		^**^	7	4.25	34.79	16.90	1.41E−11	0.04	0.18
		^**^	7	78.42	146.65	121.55	1.21E−10	0.28	0.15
		^**^	10	135.83	140.87	139.50	7.24E−10	0.38	0.28

* SNPs are significant at α_E_=0.01 except for those indicated by ^**^ (α_E_=0.05).

## Discussion

### Apache Red population

While selection for highly anthocyanin-pigmented kernels appears to have been successful given the decrease in the proportion of colorless or phlobaphene-containing kernels across generations of self-pollination (Figure 2), the presence of colorless and phlobaphene-containing kernels by the S_4_ (S_3_ pericarp) generation suggests that anthocyanin content is controlled by multiple segregating loci in this population. An alternative explanation is that some of the segregating alleles could be paramutable, spontaneously converting to paramutagenic alleles, which then heritably silence other paramutable alleles (Hollick 2017). Even in the most advanced lines from the last generations grown (S_3_s, S_4_s, and S_5_s), which were grown to bulk seed for other collaborative projects on anthocyanin-rich maize, colorless and phlobaphene containing ears were still present and had to be discarded at the time of harvest.

### Anthocyanin diversity

Previously we showed that Apache Red (AR) contains a variety of anthocyanin profiles with some containing mostly pelargonidin-derived anthocyanins, a profile not previously seen in anthocyanin-rich pericarp lines (Paulsmeyer *et al.* 2017; Chatham *et al.* 2018). We also identified AR lines with high concentrations of C-glycosyl flavones, which copigment with anthocyanins to alter color intensity, hue, and stability (Trouillas *et al.* 2016). Given the diversity and unique qualities present in this population, we self-pollinated lines of the original landrace to extract as much phenotypic diversity from the population as possible, finding a wide range of anthocyanin profiles and hues. Compared to our initial survey of Apache Red (Chatham *et al.* 2018), all anthocyanin proportion ranges presented in Table 2 had broadened. While whole kernels rather than dissected pericarp were used for this analysis, we suspect that aleurone, embryo, and other parts of the kernel contribute only minimally to overall flavone and anthocyanin content. Previously we confirmed the identity and presence of anthocyanins and flavones in AR pericarp fractions and found that pericarp profiles and whole kernel profiles were comparable (Chatham *et al.* 2018).

Compared to reports of other purple corn varieties, we observed higher percentages of acylated anthocyanins (72%), pelargonidin-derived (99%) and peonidin-derived anthocyanins (50%), and condensed forms (83%) (Table 2). Total pelargonidin-derived anthocyanins reached nearly 1400 µg/g, cyanidin-derived 872 µg/g, and peonidin-derived 274 µg/g. The lower peonidin content is consistent with our previous findings (Paulsmeyer *et al.* 2017), but peonidin content is of interest due to its potential to shift hue to a slightly more bluish-red color compared to cyanidin (Giusti and Wrolstad 2001). While cyanidin content is lower than previously reported in other pericarp-pigmented purple corn lines, the pelargonidin content is much higher. Currently, orange-red natural colorant sources are limited. Carotenoids can be used in applications requiring orange but are not water soluble and can be expensive and difficult to incorporate in some applications. Carmine, a red pigment derived from cochineal insects, also provides a true red/orange but can controversial due to its nonvegetarian source. A pelargonidin-dominant, high anthocyanin content purple corn line could provide a much-needed source of water-soluble, acid-stable plant-based red/orange colorant for the food and beverage industry (Galaffu *et al.* 2015).

### Relationship between anthocyanin profile and λ_Max_

A strong relationship between cyanidin-derived anthocyanins and λ_Max_ was expected; however, the correlations with condensed forms and with acylated forms were more surprising. Flavanol–anthocyanin condensed forms have been reported to contribute a bluish hue to wine (Dipalmo *et al.* 2016), and in purple corn extracts appeared to result in a darker red color but otherwise did not affect color (Luna-Vital *et al.* 2017). Acylated and condensed forms may also influence anthocyanin stability. Acylated anthocyanins are known to positively impact stability (Zhao *et al.* 2017), but reports on the stability of condensed forms are mixed. Some found that lines containing condensed forms produced more stable extracts (Haggard *et al.* 2018), while others showed no contribution to stability (Luna-Vital *et al.* 2017). Flavone content was also significant in modeling λ_Max_. This is consistent with previous findings (Chatham *et al.* 2019a) but is our first evidence suggesting that when co-occurring in the same line, C-glycosyl flavone and anthocyanin copigmentation is significant in the determination of the resulting extract color. This will likely have major implications on breeding objectives; that is, when an orange extract is desired, it will be essential to minimize flavone content to avoid an increase in λ_Max_ and a shift from orange to red. Yet when a red-pink or more berry-colored extract is desired, flavone content should be maximized.

While all major anthocyanin factors were significant, interpretation of these results must consider collinearity between factors. The proportion of condensed and acylated forms were negatively correlated (*r* = −0.79, *P* < 2.2e−16). Some of the condensed forms identified did include acylated groups, and while this could contribute to the correlations, acylated condensed forms were typically present in only minor amounts. This correlation suggests that acylation and polymerization to make condensed forms are competing processes. Furthermore, if either influenced λ_Max_, the correlation could result in both being significant factors. The proportion of condensed and cyanidin forms were also negatively correlated (*r* = −0.26, *P* = 5.2e−15). Likewise, this correlation could influence the significance of condensed forms in modeling λ_Max_.

Flavones were positively correlated with condensed forms (*r* = 0.39, *P* < 2.2e−16) and negatively correlated with percent cyanidin-derived (*r* = −0.50, *P* < 2.2e−16) and acylated anthocyanins (*r* = −0.29, *P* < 2.2e−16). While it is possible that these correlations are artifacts of population structure, there are also biochemical-based hypotheses that could explain them. For example, the flavanol component of condensed forms and flavones could share similar regulatory mechanisms, thus explaining their co-occurrence in many of the lines. The relationship between flavones and pelargonidin-derived anthocyanins could be explained by substrate specificity. Likewise, if *Fns1 (Flavone synthase1)*, the first enzyme in the flavone-specific branch of the pathway (see Figure 1) preferred naringenin, a correlation between flavone content and pelargonidin content would be expected, with low flavone concentrations in cyanidin dominant lines. The lack of abundant luteolin-based (eriodictyol-derived) flavones and the exclusivity of apigenin-based (naringenin-derived) flavones in AR lines would support this hypothesis. Some luteolin compounds were detected with mass spectrometry but were not present in high enough concentrations to adequately measure (Chatham *et al.* 2018). To further examine the possibility that correlations were due to genetic linkage, we plotted PC1 and PC2 scores along the axes of the kinship matrix (Supplementary Figure S3). While some evidence of a correlation between population structure and PC score was present, calculations of pairwise F_ST_ between subpopulations created based on PC1 and PC2 scores (0.006 and 0.013, respectively) indicated minimal fixation within PC groups.

Analyzing the flavonoid chemistry in Apache Red revealed that extract color is determined by more than just cyanidin-to-pelargonidin ratio. To successfully breed for specific hues, all factors should be considered in a breeding program. However, phenotyping to this extent is time-consuming and expensive. Identifying the loci responsible for various anthocyanin decorations and flavones and the ratio between them would be beneficial for designing markers for use in marker-assisted selection (MAS).

### Relationship between anthocyanin profile and Abs_max_

The correlation between flavone and anthocyanin content suggests a major regulatory factor functioning at a step prior to the branch point between anthocyanins and flavones, but the negative correlation with condensed forms lends support to a competitive zero-sum-like model of flux. Correlations with specific anthocyanin types could be an indication of substrate specificity in downstream processes or could be due to population structure. All lines here were derived from the AR landrace, making population structure an important consideration.

The relationship with flavones may be due to the absorbance increasing effect of flavone–anthocyanin copigmentation (Chatham *et al.* 2019a). Surprisingly, condensed forms and acylated forms were not correlated with Abs_max_. For condensed forms, this loss of a negative correlation (compared to total anthocyanin content) could be attributed to color-enhancing properties of condensed forms as has been seen previously in purple corn (Luna-Vital *et al.* 2017). The lack of a correlation between proportion of acylated anthocyanins and Abs_max_ despite the significant relationship seen with anthocyanin content is more difficult to explain. Acylation has not been associated with a significant increase in absorbance. However, this could be an indirect effect of the correlation that exists between acylated anthocyanins and flavones in this population. Generally, high flavone concentration was observed in lines with smaller proportions of acylated anthocyanins. These extracts could have lower acylation and lower overall anthocyanin content but not necessarily have a correspondingly low Abs_max_ due to the presence of flavones and the effects of copigmentation. Modeling anthocyanin content as a function of various flavonoid groups suggests a complex model of flux, in which each factor should be considered in the context of the others. Interactions between factors could directly influence Abs_max_ as is the case for copigmentation (Chatham *et al.* 2019a) or could be indications of the underlying biology, for example, a regulator controlling both anthocyanins and flavones.

### Genotyping and population structure

Given the likely genetic distance between Apache Red and pools of current elite maize breeding germplasm, we chose to utilize genotyping-by-sequencing (GBS) technologies. While this helps avoid ascertainment bias associated with SNP arrays and maximize the number of markers, it limits the application of these markers in other distantly related maize varieties and landraces. Since the lines used in this study were derived from the same landrace, controlling for population structure was a major concern in order to reduce false positives, yet maintain the power to detect significant associations between genotypes and traits. A PCA of the genotype data for all lines showed that PC1 explained about 10% of the variance in the dataset. A drop in the amount of variance explained by each PC (to 2%) was found between PC6 and PC7, and thus 7 PCs were used to help control for population structure in addition to the kinship matrix calculated by GAPIT (Lipka *et al.* 2012).

To further gauge the effect of population structure, we calculated F_ST_ and D between various subgroups of AR lines. Using the 15 original S_1_ lines as subgroups produced the highest values of F_ST_ and D (0.12 and 0.09, respectively) indicating these are the primary source of population structure in the AR population (Supplementary Figure S4). Hierarchical clustering using marker data produced eight subgroups with lower F_ST_ and D values (ranging 0.03 to 0.1) compared to the S_1_s; however, hierarchical clustering using phenotypes produced six clusters in which F_ST_ and D values were considerably lower (ranging from 0 to 0.01) (Supplementary Figure S4). The lack of structure among phenotypic classes lends support to hypotheses that correlations in the data are due to biological explanations other than population structure. Genome-wide linkage began to decay significantly by 1.5 Mb when calculated by GAPIT, and when a 10-site or 50-site window was used to calculate LD in TASSEL, significant decay was seen by 0.8 and 2.5 Mb, respectively with an *R*^2^ threshold of 0.1 (Supplementary Figure S5). This is indicative of the number of historical recombination events captured in this study and thus its resolution for marker–trait associations.

### Mapping pelargonidin, cyanidin, and peonidin content

As discussed above, percentages of these anthocyanidins have a significant effect on extract color, warranting the identification of responsible genetic elements and eventually the design markers for use in marker-assisted selection (MAS). In mapping the proportion of cyanidin-derived anthocyanins, we expected to find *Pr1*, a flavonoid 3’ hydroxylase (F3’H) (Sharma *et al.* 2011) in cyanidin containing aleurone lines. F3’H catalyzes the conversion of naringenin to eriodictyol and dihydrokaempferol to dihydroquercetin, the steps necessary for producing cyanidin. In aleurone-pigmented varieties, recessive *pr1/pr1* results in the accumulation of almost exclusively pelargonidin-derived anthocyanins and therefore red or pink kernels instead of the purple or blue cyanidin-containing kernels (Coe et al. 1988; Cone 2007; Sharma *et al.* 2011).

Indeed, mapping cyanidin-derived anthocyanins resulted in a highly significant peak on Chromosome 5 (Chr 5) near *Pr1* (Figure 4A, Table 4). In aleurone, three nonfunctional *pr1* alleles have been identified, including a 24 nucleotide TA repeat insertion in the 5’ upstream region, a 17 nucleotide deletion near the TATA box, and a Ds insertion in the first exon (Sharma *et al.* 2011). Cloning and sequencing of *Pr1* and *pr1* alleles in several AR lines suggests that the same 17 nucleotide deletion observed in aleurone is responsible for nonfunctional *pr1* activity in AR (data not shown).

Mapping the proportion of peonidin-derived anthocyanins produced similar results—a signal on Chr 5 near *Pr1*. The peonidin precursor, believed to be homoeriodictyol (Larson 1989), depends on the conversion of naringenin to eriodictyol catalyzed by the *Pr1* gene product (*i.e.*, *Pr1* makes the precursor (eriodictyol) to the precursor (homoeriodictyol) of peonidin). Thus, both *Pr1* and an anthocyanin *S*-adenosyl-_L_-methionine-dependent *O*-methyltransferase (AOMT) should be required for synthesis of homoeriodictyol and thus peonidin. To isolate AOMT action, a variable describing the proportion of all eriodictyol-based compounds that were converted to homoeriodictyol-based compounds was used. This was calculated by dividing the total peonidin content by the sum of cyanidin and peonidin content. When mapping this variable (Figure 4B), markers near *Pr1* were still significant, but a signal on Chr 4 around 200 Mb emerged. Individual chromosome Manhattan plots showing loci of interest can be found in Supplementary Figure S6, A and B.

### Methyltransferase candidates

Many AOMTs have been identified in plants producing O-methoxy anthocyanidins in dicots, but no true anthocyanin-specific AOMTs have been identified in monocots (Provenzano *et al.* 2014). Recently, a CcoAOMT was identified in wheat (*Triticum aestivum*) through transcriptome analysis of purple pericarp. *TaCcoAOMT* was upregulated in peonidin producing wheat pericarp, but the locus was not further characterized (Liu *et al.* 2016). No methyltransferase with AOMT activity responsible for peonidin biosynthesis in maize has been identified, but the presence of peonidin-derived anthocyanins suggests it or an enzyme with similar function exists. Amino acid sequences of AOMTs from *Vitis vinifera* and *Glycine max* were blasted against the maize genome (Jiao *et al.* 2017) to identify a list of potential candidates (Supplementary Table S1). To narrow down the likely candidates, amino acid sequences of AOMTs, CcoAOMTs, and caffeic acid OMTs from other species were aligned with the maize candidates and a maximum likelihood phylogeny was created (Supplementary Figure S7). A clear outgroup containing the caffeic acid OMTs is evident, excluding Zm00001d052683 and Zm0001d052684. Zm00001d052843 and Zm00001d024596 were also in outgroups apart from the remaining AOMTs and CcoAOMTs, which clustered into two groups. Zm00001d045206 and Zm00001d036293 clustered with the CcoAOMTs, but are located on Chr 9 and Chr 6, respectively. The remaining candidates, Zm00001d052841 and Zm00001d052842, were the next most closely related candidates to the AOMTs and CcoAOMTs, are identified as putative CcoAOMTs, and are located on Chr 4 around 202 Mb.

The methyltransferase phylogeny and the proximity to the signal mapped to Chr 4 for peonidin content suggest Zm00001d052841 and Zm00001d052842 are likely candidates. These candidates were aligned to several methyltransferases with known AOMT and CcoAOMT activity, as well as the CcoAOMT expressed in purple wheat pericarp (Supplementary Figure S8). Amino acid residues associated with enzyme function were conserved. Variability was observed between sequences for Arg217 (CoA binding), Tyr219, and Tyr223. Previously, modeling of substrate binding showed that the bulky tyrosine residues present in CcoAOMTs in this location clash with the anthocyanin 3-glucose moiety, that is, the site cannot accommodate the larger anthocyanin 3-glucose moiety due to obstruction by a bulky tyrosine (Provenzano *et al.* 2014). These tyrosines are substituted with the smaller amino acids glycine and leucine in Petunia AOMTS, with arginine and leucine in *TaCcoAOMT*, and with isoleucine, leucine, and phenylalanine in the two maize candidates. Replacement with phenylalanine certainly would not reduce size or bulkiness to the same degree as a glycine or leucine substitution, but a tyrosine-to-phenylalanine conversion at one of these locations (Tyr219 or Tyr223) was observed for VvAOMT1, VvAOMT3, DhAOMT, and GmAOMT, all of which have anthocyanin OMT activity. Moreover, the relatively low proportions of peonidin (max = 50%) compared to cyanidin (max = 86%) observed here and in purple wheat (Abdel-Aal *et al.* 2006) as well as in other purple corn studies (Paulsmeyer *et al.* 2017) suggest that the responsible enzyme has relatively low efficiency. If CcoAOMTs were co-opted for anthocyanin use, it is reasonable that they would have suboptimal performance compared to the AOMTs specialized for peonidin production. Here, we found higher proportions of peonidin-derived anthocyanins when anthocyanin content was low (*r* = −0.24, *P* < 2.2e−16), lending support for this hypothesis.

While these residue changes suggest that Zm00001d052841 and Zm00001d052842 could better accommodate the anthocyanin-3-glucoside moiety than the canonical maize CcoAOMTs, it is believed that the methyltransferase in maize acts prior to glycosylation (Larson 1989). This is evidenced by an S-adenosylmethionine-flavonoid 3ʹ-O-methyltransferase capable of utilizing eriodictyol, luteolin, and quercetin as substrates, but not quercetin 3-glucoside in crude protein extracts from maize seedlings, leaf sheath, and aleurone (Larson 1989). Comparing Zm00001d052841 and Zm00001d052842 shows that the two are highly similar except for several amino acids and an extra string of 33 amino acids at the beginning of the Zm00001d052841 sequence. Further studies will be required to determine which is responsible for AOMT-like activity, what substrates can be accommodated by the product, and the residues most important for enzyme efficiency.

#### Mapping anthocyanin decorations

*Acylated anthocyanins:* Wide ranges of acylated anthocyanins, flavanol–anthocyanin condensed forms, and flavones were observed in the phenotypic dataset. Acylated and condensed forms had somewhat bimodal distributions (Figure 3), suggesting they are simply inherited. Mapping acylated anthocyanin proportions (Figure 4C) produced a peak on Chr 1 near 306 Mb, which aligns with Aat1, the anthocyanin acyltransferase recently identified in aleurone-pigmented lines and located at 305.7 Mb. This helps confirm that Aat1 is associated with acylated anthocyanin content in pericarp as well as in aleurone. While this was by far the most significant SNP for acylated anthocyanins, several other significant loci were also found and are listed in Table 4.

*Flavanol–anthocyanin condensed forms:* Mapping produced a wide signal surrounding P1, a known MYB regulator of phlobaphene and flavone biosynthesis (Morohashi et al. 2012, p. 1) (Figure 4D). We also observed signals on Chr 9 near Fns1 (130.6 Mb), which encodes a flavanone 2-hydroxylase required for flavone biosynthesis. Given the correlation between flavones and condensed forms, finding these loci was not surprising.

A signal near *Pl1* (Chr 6 at 113 Mb), the MYB required for anthocyanin biosynthesis in maize plant tissue, was also found. Unsurprisingly, this signal disappeared when mapping condensed forms as a proportion of the total anthocyanin content, suggesting a role in anthocyanin concentrations rather than the presence of, or proportion of, condensed forms. A locus (Zm00001d005106) with similarity to *Rif1* was found 3 Mb from the signal observed on Chr 2 at 161.59 Mb. The signal on Chr 4 (195.24 Mb) is close to *C2* (*Colorless2*; Zm00001d052673, 196.89 Mb), a chalcone synthase (CHS) directly activated by *P1.* C2 is the first committed step into the pathway for both anthocyanins and flavones (Figure 1) and has been detected previously as quantitative trait loci (QTLs) associated with maysin content (Meyer *et al.* 2007). No promising candidates were found for the signal on Chr 2 at 130.57 Mb.

Mapping the proportion of condensed forms produced several signals that were redundant with previous mapping results, including signals near *P1* and *Fns1. Aat1* was also previously identified for the proportion of acylated anthocyanins but given the inverse relationship between the proportion of acylated and condensed forms, its recurrence here is unsurprising. New signals emerged on Chr1, Chr 3, and Chr 5, as well as a signal on Chr 4 that was significant only at the α_E_ = 0.05 threshold. The peak on Chr1 is close to *Aat1*, which was detected previously when mapping acylated anthocyanins. This was likely a result of an inverse correlation between condensed forms and acylated anthocyanins (Figure 3). Another signal on Chr 1 was found for both total and percent condensed forms near *Chi1 (Chalcone isomerase1* 298.6 Mb) (Grotewold and Peterson 1994), a flavonoid biosynthesis structural gene shared by both condensed forms and flavones. The peak observed for percent condensed forms had its most significant SNP about 3 Mb from CHI1; however, the range of significant SNPs stretches through the end of the chromosome likely due to the proximally located *Aat1.* No other promising candidates closer to the most significant SNP were identified.

The significant SNP on Chr 4 was found at 176.6 Mb for which the best candidate identified for was a blast hit for a *TT13*, an *Arabidopsis* tonoplast-located ATPase required for proanthocyanidin accumulation in vacuoles. *TT13* in combination with *TT12*, a MATE flavonoid transporter, is required for epicatechin (proanthocyanidin precursor) transport into the vacuole (Appelhagen *et al.* 2015). Since both proanthocyanidins and condensed forms require flavan-3-ols as building blocks, it is plausible that an ATPase in maize could be required for effective transport of flavanols and thus the synthesis of condensed forms (Chatham et al. 2019b). The best candidate for the signal on Chr 5 was a UDP-D-glucose dehydrogenase located at 6.95 Mb.

To further investigate factors that might be controlling flux toward the formation of condensed forms, we converted all identified compounds to moles and calculated condensed forms as a molar proportion of total measurable flux into the phenylpropanoid pathway. We again found signals near *P1* and observed a strong signal near *pr1.* New signals were found on Chr 2, Chr 3, and Chr 4. All but the signal on Chr 2 were significant only at the less stringent α_E_=0.05 (Table 4).

### Mapping flavone content

C-glycosyl flavones can largely influence anthocyanin extract hue and intensity and are therefore an important consideration in a breeding program aiming to optimize hue (Chatham *et al.* 2019a). They are also the precursors to maysin, a C-glycosyl flavone that provides resistance to corn earworm (*Helicoverpa zea*) (Byrne *et al.* 1996). Surprisingly, we were not able to identify significant quantities of maysin in the AR population, suggesting fixation of a null *Sm1* (*Salmon silks1)* or *Sm2* (*Salmon silks2)* allele. *Sm1* and *Sm2* encode the rhamnose synthase and rhamnose transferase that convert *C*-hexosyl flavones into maysin or apimaysin (Casas *et al.* 2016) (Figure 1). As determined by HPLC and confirmed using mass spectrometry (Chatham *et al.* 2018), the major C-glycosyl flavones present in AR lines were di-*C*, *C*-hexosyl apigenin, *C*-hexosyl-*C*-pentosyl apigenin, *C*-pentosyl-*C*-hexosyl apigenin, and *C*-hexosyl apigenin.

Mapping flavone content resulted in many of the same loci that were detected for condensed forms. As expected, a large signal near *P1* was identified (Figure 4G). However, the signal was wide, ranging from 27 to 53 Mb with the most significant SNP around 39 Mb, more than 9 Mb away from *P1* (Supplementary Figure S9). Several loci involved in sugar metabolism are near *P1* and are possibly regulated by it (Morohashi *et al.* 2012). This signal could represent several important loci, but linkage blocks in this area could also be contributing to the signal width (Supplementary Figure S10). In addition to this major locus, a signal was identified on Chr 1 at 294 Mb, about 4.5 Mb from *Chi1*, which encodes a chalcone isomerase that functions prior to the branch point in the pathway for flavones or anthocyanins. Given the correlation between flavones and condensed forms, another potential candidate could be an ANR/LAR blast match located at 290.53 Mb (Zm00001d034357).

We also found a signal near *Fns1* (Chr 9, 130.65 Mb), the flavone synthase required for flavone biosynthesis in maize (Ferreyra *et al.* 2015), and a signal near *Pr1* (Chr5 184.49 Mb). Most flavones detected in AR were apigenin-derived, containing a single hydroxyl group on the B-ring. Rather than an inability to produce luteolin compounds (two hydroxyl groups on the B-ring) though, we suspect this is more likely due to trait linkage resulting from a fixed recessive *pr1* in AR families that produced large quantities of flavones. Intercrossing between *Pr1/-* lines (evident by cyanidin-dominant anthocyanins) and high flavone lines would likely produce luteolin-derived flavones; however, this should be tested to confirm. Other signals identified when mapping flavones, but without promising candidates, are listed in Table 4.

With data quantifying different flavone species from HPLC analysis, we mapped the proportion of each flavone type relative to total flavone content. We again identified a signal near *Fns1*, as well as one on Chr 6 near *Cgt1*, a dual *C-/O-*glycosyltransferase implicated in the formation of *C-*hexosyl flavones (Ferreyra *et al.* 2013). *Cgt1* is capable of *C-*glycosylating at both the 6-*C* and 8-*C* positions, but this is the first indication of its role in creating di-*C, C-*hexosyl flavones, glycosylated at both positions on the same molecule. While it is possible that *Cgt1* is also capable of *C-*glycosylating pentose moieties, it is difficult to separate the functions in this dataset as no *C-*pentosyl flavones were identified that did not also contain a hexose. In addition to these signals, peaks were found on Chr 5, 7, and 8, which are listed in Table 4. No obvious candidates were identified for these, but several plausible loci were considered. For the peak on Chr 7 with the most significant SNP located at 144.83 Mb and spanning 138–146 Mb, two glycosyltransferases were found around 144 Mb (Zm00001d021167 and Zm00001d021168). This signal is also close to *myb152* (148.15 Mb, Zm00001d021296) a positive regulator of the phenylpropanoid pathway (Agarwal *et al.* 2016). We also found a MYB transcription factor close to the signal on Chr 8 (*myb147*, Zm00001d010933) as well as a flavonoid 3’-hydroxylase (Zm00001d010521) orthologous to *Transparent Testa7* (*TT7*), which functions similarly to *Pr1* in maize, determining the ratio of kaempferol to quercetin in Arabidopsis seed coats (Schoenbohm *et al.* 2000). Variations in flavone structure could influence copigmentation efficacy (Trouillas *et al.* 2016); however, more information is needed before specific breeding objectives for optimal flavone content can be defined.

### Mapping anthocyanin concentration and Abs_max_

Given the differences in correlations with different flavonoid profiles, we expected slightly different mapping results for anthocyanin content and Abs_max_. Each produced highly significant signals on Chr 1 near *Aat1*, with the most significant SNP less than 1 Mb away, within the range of LD for this population. This suggests that despite being a structural gene, acylation by *Aat1* is important for achieving highly pigmented extracts. In *V. vinifera¸* MATE transporters were identified that selectively transported acylated anthocyanins into the vacuole compared to their unacylated forms (Gomez *et al.* 2009). While this offers a plausible explanation for a highly significant peak near *Aat1*, no anthocyanin-transporting MATEs have been identified in maize and this phenomenon has not been associated with the GST/ABC transporter system (*Bz2/Mrp3* in maize).

Examining the percent of acylation in Figure 3 and Supplementary Figure S2 shows a bimodal distribution, with the lower acylation group having both fewer observations and a smaller spread of anthocyanin content and Abs_max._ This association could be due to linkage between *Aat1* and some other factor related to anthocyanin content, but LD in the surrounding region suggests this is unlikely (Supplementary Figure S10). Nonetheless, to account for potential linkage, mapping was performed using acylation as a covariate, and modeling *Aat1* as a fixed factor, but the signal was still present. To overcome this, the tails of the acylation proportion distribution—less than 0.35 and greater than 0.75—were removed from the data to isolate anthocyanin content.

Using the truncated dataset for mapping (acyl proportion tails removed, leaving 84% of the original AR panel) eliminated the signal near *Aat1* as well as several other smaller signals (Figure 5). Examining these eliminated loci could reveal factors associated uniquely with acylation or factors that improve the trafficking and storage efficiency of acylated anthocyanins if maize follows a pattern similar to that described in *V. vinifera* (Gomez *et al.* 2009). These signals include Chr 2 at 150.38 Mb, Chr 5 at 8.56 Mb, Chr 6 at 21.26 Mb, and Chr 9 at 143.53 Mb for anthocyanin content, and Chr 4 at 23.41 Mb for Abs_max_. All but the signal on Chr 5 had low minor allele frequencies (0.05–0.07), and all but the signal on Chr 6 consisted of a single significant marker. No particularly promising candidates were identified for any of these signals, but the closest candidates considered were a potential MATE transporter located on Chr 2 at 154.1 Mb (Zm00001d005018) and a UDP-D-glucose dehydrogenase on Chr 5 at 6.95 Mb involved in sugar metabolism and expressed in pericarp of both *P1-rr* (phlobaphene rich) and *P1-ww* (no phlobaphene) lines (Morohashi *et al.* 2012). Another potential candidate is *Chi3* (*Chalcone isomerase3*), located at 2.6 Mb*.* A signal at this same location on Chr 5 was previously identified for the proportion of condensed forms.

Given previous studies on anthocyanin regulation in maize tissues and previous reports breeding purple corn with anthocyanin-rich pericarp (Lago *et al.* 2014a, 2014b), we expected mapping to produce signals near *B1*, the plant color-associated bHLH, and *Pl1*, the plant color-associated MYB. While SNPs close to *Pl1* were found, we did not identify any significant signals near *B1.* Instead, SNPs proximal to *R1* (less than 1 Mb away) were found for both anthocyanin content and Abs_max_ using both the complete and truncated sets of lines. The *R1* locus is diverse, and while usually associated with aleurone color, some alleles of *R1* (*e.g.*, *R1-ch*), and its neighboring (or allelic) loci *Sn1* (*Scutellar node1*) (Tonelli *et al.* 1991), *Lc1* (*Leaf color1)* (Dooner and Kermicle 1976), and *Hopi* (Petroni *et al.* 2000) can produce anthocyanins in pericarp (Portwood *et al.* 2019). Additional research will be required to identify the specific bHLH allele in AR and whether it interacts with other proteins in the manner described previously for aleurone-pigmented lines.

*R1* is a bHLH transcription factor and part of the MBW ternary regulatory complex associated with anthocyanin biosynthesis across species (Lloyd *et al.* 2017). Therefore, we expected to also find a WD40 as well, but no signal was found near *Pac1* (Chr 5 at 201.8 Mb), the WD repeat protein component of the MBW regulatory complex. *R1* has also been shown to interact with *Rif1* (*R-interacting factor1;* Chr 7 at 76.6 Mb), potentially to regulate control of early *versus* late biosynthetic steps in the anthocyanin pathway (Kong *et al.* 2012). Several signals were consistently found on Chr 7, with one stretching to 78.42 for Abs_max_ when using the truncated set of lines. However, this same SNP was not significant for the other three analyses. Key components of the regulatory complex such as these could be fixed in the Apache Red population. Alternatively, pericarp-specific genes could exist that have not yet been identified and for which this study did not have the power to detect.

A major signal was consistently identified at the beginning of Chr 7, with the most significant SNP located at 16.90 Mb for Abs_max_ and at 22.61 Mb for anthocyanin content. This is close to *In1* (Chr 7 at 20.11 Mb), a recessive intensifier of anthocyanin biosynthesis in aleurone-pigmented lines (Supplementary Figure S11). *In1* shares homology to the bHLH members of the MBW regulatory complex resulting in competitive inhibition of *R1* (Burr *et al.* 1996)*.* To our knowledge, there have been no reports of *In1* functioning in pericarp, but given our results suggesting AR lines utilize *R1* instead of *B1*, it would not be surprising for *In1* to compete with *R1* in pericarp in a manner similar to that in aleurone. Not all Apache Red lines in the population were tested to determine whether spurious aleurone pigmentation had developed some time during population development, and thus it is possible that some lines may have had dilute aleurone pigmentation in addition to pericarp pigmentation. However, the contribution to total anthocyanin content would likely be minimal and not enough to cause an aleurone-only locus to be detected during mapping. With the established capacity of *in1* to significantly increase anthocyanin content in aleurone (Reddy and Peterson 1978), the *In1* locus should be explored further for maximizing anthocyanin content in pericarp.

Several signals spanning a large portion of the middle of Chr 7 were also detected repeatedly. Given the wide range of the signals which varied between analyses, it is difficult to determine the number of loci being tagged. Two potential candidates were identified, including myb152 (Zm00001d021296, 148.15 Mb), a known positive regulator of phenylpropanoid metabolism in maize, and myb162 (Zm00001d020457, 115.74 Mb), a MYB with similarity to those functioning in anthocyanin metabolism in other species (Supplementary Figure S12).

To identify factors controlling flux through the pathway, concentrations of individual anthocyanin and flavone compounds were converted from μg/ml to mols/ml, and the proportion of the total molar flux into the pathway was calculated and mapped for anthocyanins and flavones (Figure 5). Major signals were found close to *P1, Aat1*, and *Pl1.* While *R1* showed up consistently when mapping anthocyanin content overall, it was not detectable when looking at proportions of flux through the pathway. This suggests *Pl1* may play a more significant role in determining flux through the pathway, influencing the ratio of flavones and anthocyanins, while *R1* may contribute more to overall content. The peak at the beginning of Chr 1 was close to *P1* but not nearly as close as other candidates have been to their assumed loci (*e.g.*, *Aat1*, *Pl1, R1*). *P1* is located at 48 Mb, but the most significant SNP for anthocyanin/flavone flux was found at 35 Mb. Higher linkage in these areas could possibly be contributing to the gap between the most significant SNPs and *P1* (Supplementary Figure S4).

Mapping results for the molar proportion of anthocyanins were similar to mapping total concentrations and Abs_max_, but one new QTL was identified on Chr 7. This single SNP was only significant at the stringent α_E_=0.05 Bonferroni correction threshold and was found at 156.4 Mb. This is close to a potential ortholog of the *Arabidopsis TT12 (Transparent Testa12*) MATE transporter of flavonoids (Zm00001d021629, 158.6 Mb) (Debeaujon *et al.* 2001) and other flavonoid transporting MATEs in other species (Supplementary Figure S3) (Gomez *et al.* 2009; Zhao *et al.* 2011; M’mbone *et al.* 2018). This is particularly interesting given the highly significant effect of SNPs near *Aat1* for both competitive flux and overall flux (Figures 4 and 5)*. Vv*AM1, *Vv*AM3, and *Mt*MATE2 all preferentially transport acylated anthocyanins (Gomez *et al.* 2009; Zhao *et al.* 2011), and MtMATE1 is specific to epicatechin (Zhao and Dixon 2009; Zhao *et al.* 2011)*.* A candidate MATE with specificity for acylated anthocyanins could explain how *Aat1* appears to contribute so significantly to anthocyanin content. A phylogeny of candidate MATEs from maize and MATEs associated with flavonoid transport in other species can be found in Supplementary Figure S13. Differences in specificity could also play a role in the relative amounts of condensed forms *versus* anthocyanins that are stored in the vacuole.

### Applications

Highly concentrated purple pericarp is only found in a select portion of maize germplasm, most of which are not adapted to a Midwest environment and do not have yields comparable to today’s hybrids. Apache Red, a pericarp-pigmented purple corn variety, offers high anthocyanin concentration and variability in extract colors (Chatham *et al.* 2018). However, due to linkage drag from donor purple corn parents, many generations of backcrossing to elite, adapted inbreds (as the recurrent parent) may be required to recover desired agronomic traits while maintaining anthocyanin concentrations. Furthermore, as different desirable traits are identified from the donor (*i.e.*, different anthocyanin types/decorations), additional introgression pipelines may be needed.

The development of separate flavone-rich lines and anthocyanin-rich lines is warranted to allow for the maximum range of applications. Ideally, a pelargonidin-rich line producing orange extract, a cyanidin-rich line producing red-pink extract, and a colorless line rich in C-glycosyl flavones with the greatest capacity for copigmentation would be bred. Doing this would allow a manufacturer to design a range of hues for use in food and beverage products by mixing different ratios of the pelargonidin extract, cyanidin extract, and flavone extract, as described previously (Chatham *et al.* 2019a). Anthocyanin-rich lines should be further optimized to maximize stability and anthocyanin content. Acylated anthocyanins should be maximized, while condensed forms should be minimized. Reports of improved stability from condensed forms are sparse, and condensed forms were associated with flavone content in this population, something that should be minimized in breeding anthocyanin lines. Condensed forms could also represent a reduction in overall content, since two units (1 flavan-3-ol + 1 anthocyanin) are required to make one condensed form.

The development of markers for the candidates and known genes identified herein to be associated with anthocyanin type and decoration could improve selection efficiency. For example, pelargonidin-rich lines could be selected at the seedling stage by choosing *pr1/pr1* lines and could be tracked through backcrossing by differentiating between *Pr1/pr1* and *Pr1/Pr1* individuals to avoid subsequent test crosses ensuring maintenance of the recessive allele. Combined with markers for *Pr1*, the identification of a methyltransferase candidate for peonidin content could enable the development of markers that aid in creating peonidin-dominant lines (requiring both *Pr1/-* and a functional *AOMT* due to their complimentary gene action). Based on results presented here, purple corn breeding programs should select for nonfunctional *p1* and nonfunctional ANR or LAR candidates to eliminate flavones and condensed forms while selecting for functional *Aat1* to maintain acylated anthocyanins.

**Figure 6:**
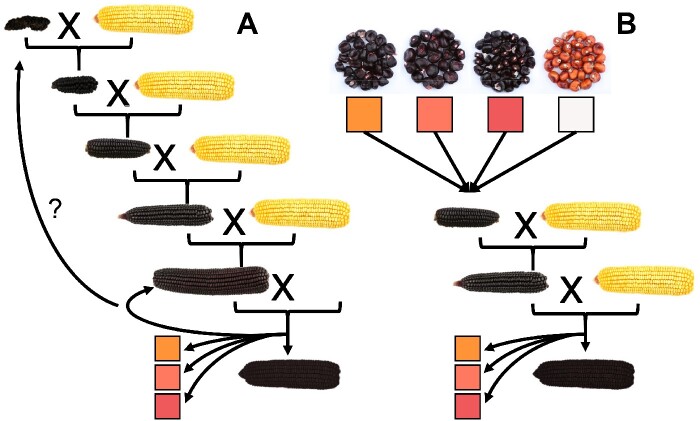
Traditional introgression (A) compared to landrace improvement (B). When multiple traits are required from a landrace, improvement of landrace materials prior to introgression may reduce linkage drag and the number of backcrosses required to regain necessary agronomic traits while maintaining the landrace sourced trait.

Pre-breeding of landraces like AR or other unadapted germplasm could make desirable traits more accessible by helping to eliminate deleterious alleles that may later be “dragged” via linkage during backcrossing (Gorjanc *et al.* 2016). Figure 6 illustrates the benefits of a landrace pre-breeding scheme in which the number of required backcross generations is reduced compared to an approach using a direct landrace by elite parent cross (Figure 6A). Knowledge of the key factors associated with both maximizing and optimizing anthocyanin content could help ensure their fixation in the development of superior donor parents. This could improve the efficiency of introgression, maximizing the transfer of desirable traits while reducing movement of those that are undesirable. Lastly, superior donor parents could streamline the introgression process as breeding goals shift or change, saving the breeder from having to return to unadapted sources.

## Supplementary Material

jkaa062_Supplementary_Data
