# Supplementary material for: Linking anthocyanin diversity, hue, and genetics in purple corn
Source: G3 (Bethesda). 2021 Jan 11;11(2):jkaa062. doi: 10.1093/g3journal/jkaa062 (PMC8022952; doi:10.1093/g3journal/jkaa062)
Supplement: jkaa062_Supplementary_Data [file jkaa062_supplementary_data.zip › Supplementary Figure Legends.docx]

**SUPPLEMENTARY FIGURES**

**Supplementary Figure 1:** Schematic for the creation of Apache Red lines.

**Supplementary Figure 2:** HPLC chromatogram example showing the quantified anthocyanin peaks, as listed in Table 3 (A); Correlations between anthocyanin composition factors and wavelength at maximum absorbance (λ_Max_), a proxy for hue (B); effects of anthocyanin composition on maximum absorbance (Abs_Max_), a proxy for color intensity. Anthocyanin content was square root transformed from μg/ml, and flavone content (μg/ml) and condensed form proportion were log transformed.

**Supplementary Figure 3:** Heatmaps of the K matrix showing relatedness between individuals and their principal component scores for anthocyanin / flavone content as shown in Figure 3. A, lines ordered according to relatedness with PC1 scores illustrated on the X axis and PC2 scores on the Y axis. B, lines in K matrix ordered according to PC1 scores or PC2 scores (C)

**Supplementary Figure 4:** Pairwise F_ST_ (bottom half of matrix) and D (top half of matrix) for each S1 family used in creating the AR population (A). Hierarchical clustering of AR lines based on marker data (B), the number of S1 families in each cluster (C), and F_ST_ (bottom half) and D (top half) for each cluster.

**Supplementary Figure 5:** LD decay using r^2^ with either a 10 site window (A) or 50 site window (B)

**Supplementary Figure 6:** Individual chromosome plots for major signals identified for the proportion of cyanidin (A), peonidin (B), and acylated anthocyanins (C). Chromosome numbers are listed in the x-axis label for each plot.

**Supplementary Figure 7:** Phylogram of plant O-methyltransferases. Amino acid sequences were aligned and maximum likelihood tree was created. Branch numbers represent bootstrap values based on 100 replicates. Sequences correspond to table 4, and Zea mays sequences are highlighted in blue.

**Supplementary Figure 8:** Alignment of Caffeoyl-CoA methyltransferases (□), Anthocyanin O-methyltransferases(◊), and candidate Zea Mays methyltransferases(*). Purple boxes and labels indicate Caffeoyl moiety binding, blue indicates CoA binding, and gray indicates SAM binding.

**Supplementary Figure 9:** Individual chromosome plots for major signals identified for flavone content (A-D), and the proportion of *C* -hexosyl- *C* -pentosyl apigenin (E-G). Chromosome numbers are listed in the x-axis label for each plot.

**Supplementary Figure 10:** Pairwise LD for chromosome 1 in the regions surrounding *P1* (A) and *Aat1* (B). SNPs labeled in green were highly significant SNPs from AR GWAS and the SNPs labeled in dark purple indicate the SNP closest to the locus of interest*.*

**Supplementary Figure 11:** Individual chromosome plots for major signals identified for total anthocyanin content in the full AR panel (A) and truncated (B).

**Supplementary Figure 12:** Phylogram of MYBs. Red labels indicate negative regulation while green labels indicate positive regulation of anthocyanin or flavonoid biosynthesis. Maize MYBs with unknown function are colored in blue. Sequences correspond to Supplementary Table 3, and branch numbers represent bootstrap values based on 100 replicates.

**Supplementary Figure 13:** Maximum likelihood phylogeny based on peptide sequences of flavonoid transporting MATEs and candidate MATEs from maize. Branch numbers represent bootstrap values based on 100 replicates.
