# Supplementary material for: Linking anthocyanin diversity, hue, and genetics in purple corn
Source: G3 (Bethesda). 2021 Jan 11;11(2):jkaa062. doi: 10.1093/g3journal/jkaa062 (PMC8022952; doi:10.1093/g3journal/jkaa062)
Supplement: jkaa062_Supplementary_Data [file jkaa062_supplementary_data.zip › Supplementary Figure S1.pptx]

## Slide 1
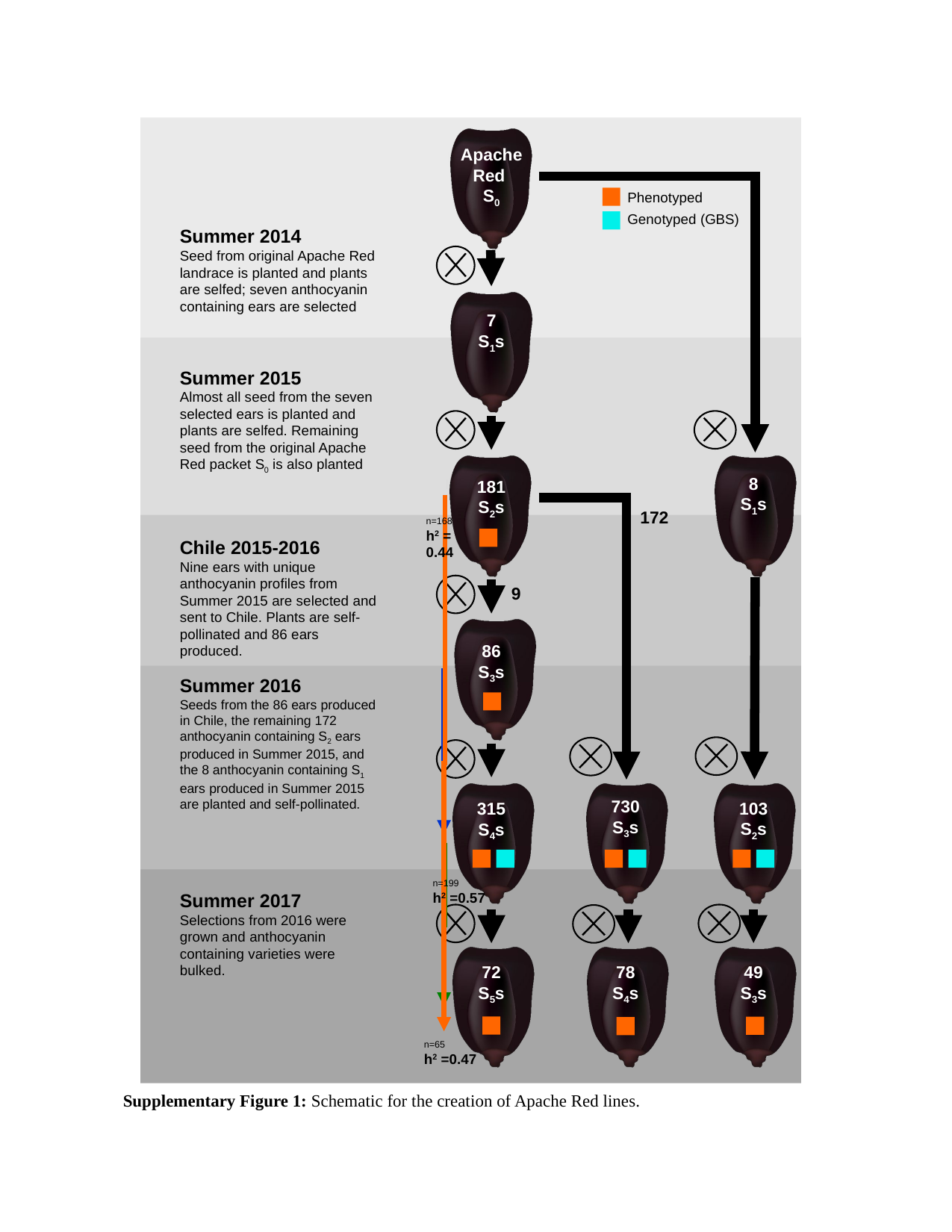

Apache Red
S0
Phenotyped
Genotyped (GBS)
Summer 2014
Seed from original Apache Red landrace is planted and plants are selfed; seven anthocyanin containing ears are selected
7
S1s
Summer 2015
Almost all seed from the seven selected ears is planted and plants are selfed. Remaining seed from the original Apache Red packet S0 is also planted
181
S2s
8
S1s
172
n=168
h2 =
0.44
Chile 2015-2016
Nine ears with unique anthocyanin profiles from Summer 2015 are selected and sent to Chile. Plants are self-pollinated and 86 ears produced.
9
86
S3s
Summer 2016
Seeds from the 86 ears produced in Chile, the remaining 172 anthocyanin containing S2 ears produced in Summer 2015, and the 8 anthocyanin containing S1 ears produced in Summer 2015 are planted and self-pollinated.
315
S4s
730
S3s
103
S2s
n=199
h2 =0.57
Summer 2017
Selections from 2016 were grown and anthocyanin containing varieties were bulked.
72
S5s
78
S4s
49
S3s
n=65
h2 =0.47
Supplementary Figure 1: Schematic for the creation of Apache Red lines.
