# Supplementary material for: Linking anthocyanin diversity, hue, and genetics in purple corn
Source: G3 (Bethesda). 2021 Jan 11;11(2):jkaa062. doi: 10.1093/g3journal/jkaa062 (PMC8022952; doi:10.1093/g3journal/jkaa062)
Supplement: jkaa062_Supplementary_Data [file jkaa062_supplementary_data.zip › Supplementary Figure S2.pptx]

## Slide 1
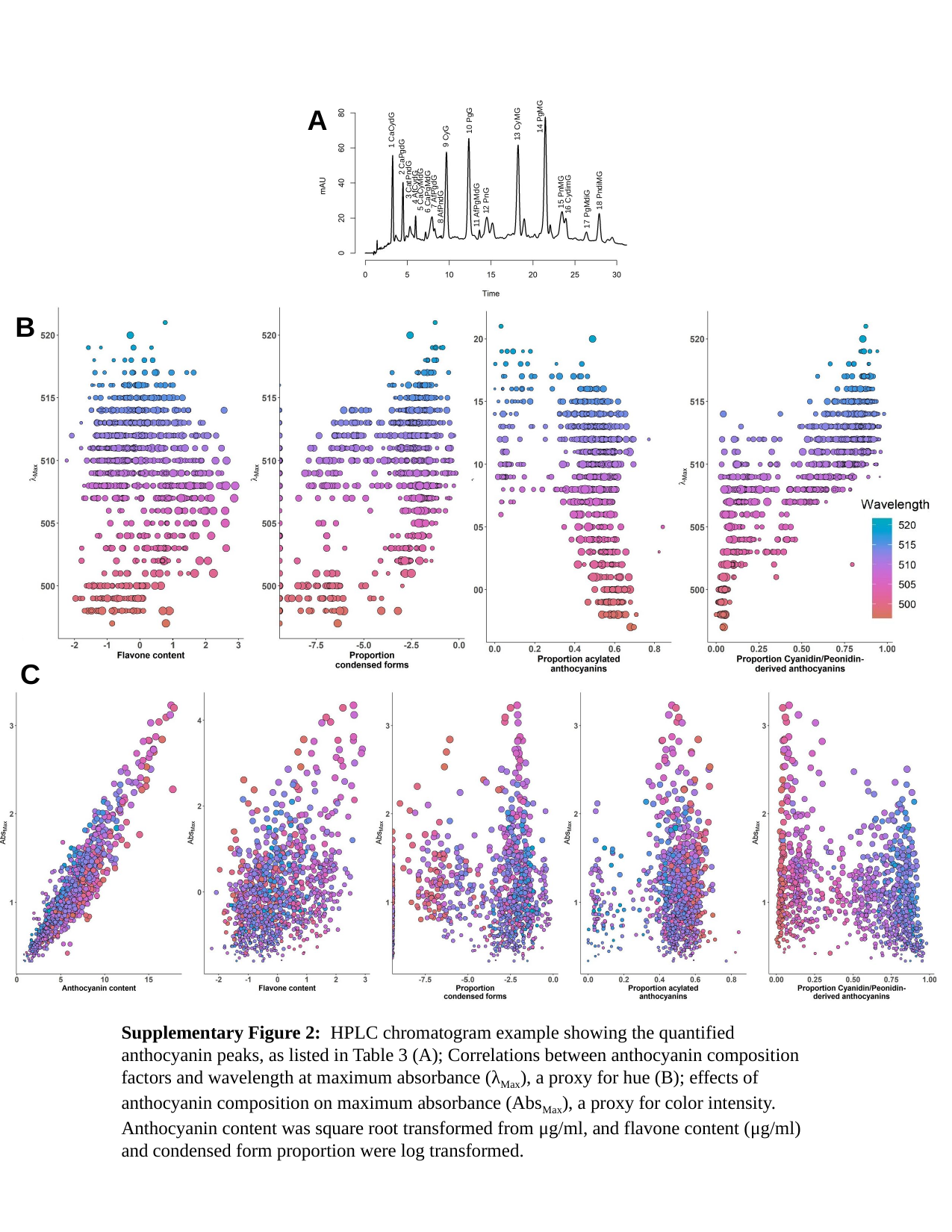

14 PgMG
10 PgG
13 CyMG
9 CyG
1 CaCydG
2 CaPgdG
3 CatPndG
4 AfCydG
15 PnMG
7 AfPgdG
18 PndiMG
5 CaCyMdG
6 CaPgMdG
12 PnG
16 CydimG
8 AfPndG
11 AfPgMdG
17 PgMdiG
A
B
C
Supplementary Figure 2: HPLC chromatogram example showing the quantified anthocyanin peaks, as listed in Table 3 (A); Correlations between anthocyanin composition factors and wavelength at maximum absorbance (λMax), a proxy for hue (B); effects of anthocyanin composition on maximum absorbance (AbsMax), a proxy for color intensity. Anthocyanin content was square root transformed from μg/ml, and flavone content (μg/ml) and condensed form proportion were log transformed.
