# Supplementary material for: Linking anthocyanin diversity, hue, and genetics in purple corn
Source: G3 (Bethesda). 2021 Jan 11;11(2):jkaa062. doi: 10.1093/g3journal/jkaa062 (PMC8022952; doi:10.1093/g3journal/jkaa062)
Supplement: jkaa062_Supplementary_Data [file jkaa062_supplementary_data.zip › Supplementary Figure S4.pptx]

## Slide 1
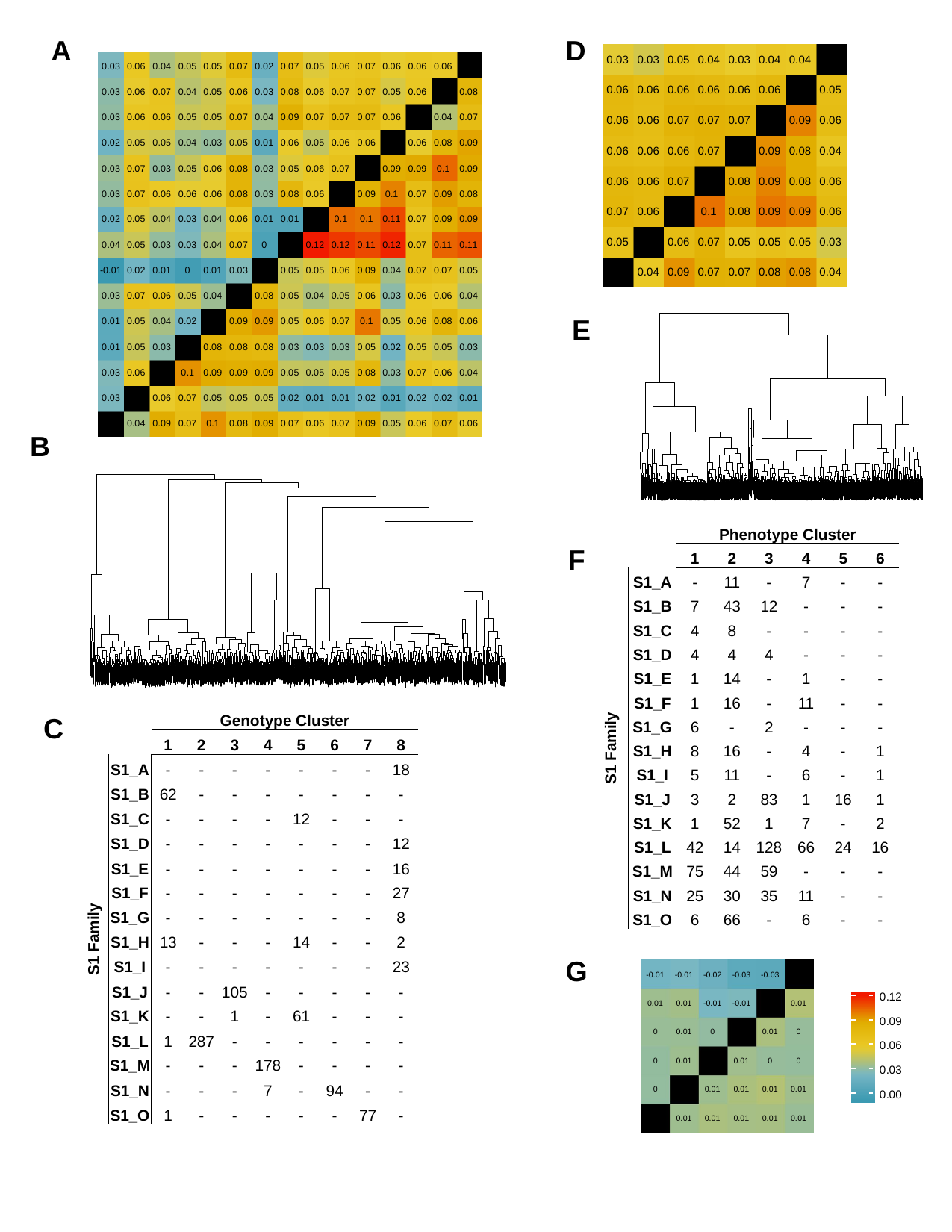

D
A
8
7
6
5
4
3
2
1
S1_O
S1_N
S1_M
S1_L
S1_K
S1_J
S1_I
S1_H
S1_G
S1_F
S1_E
S1_D
S1_C
S1_B
S1_A
E
B
| | | Phenotype Cluster | | | | | |
| --- | --- | --- | --- | --- | --- | --- | --- |
| | | 1 | 2 | 3 | 4 | 5 | 6 |
| S1 Family | S1\_A | - | 11 | - | 7 | - | - |
| | S1\_B | 7 | 43 | 12 | - | - | - |
| | S1\_C | 4 | 8 | - | - | - | - |
| | S1\_D | 4 | 4 | 4 | - | - | - |
| | S1\_E | 1 | 14 | - | 1 | - | - |
| | S1\_F | 1 | 16 | - | 11 | - | - |
| | S1\_G | 6 | - | 2 | - | - | - |
| | S1\_H | 8 | 16 | - | 4 | - | 1 |
| | S1\_I | 5 | 11 | - | 6 | - | 1 |
| | S1\_J | 3 | 2 | 83 | 1 | 16 | 1 |
| | S1\_K | 1 | 52 | 1 | 7 | - | 2 |
| | S1\_L | 42 | 14 | 128 | 66 | 24 | 16 |
| | S1\_M | 75 | 44 | 59 | - | - | - |
| | S1\_N | 25 | 30 | 35 | 11 | - | - |
| | S1\_O | 6 | 66 | - | 6 | - | - |
F
| | | Genotype Cluster | | | | | | | |
| --- | --- | --- | --- | --- | --- | --- | --- | --- | --- |
| | | 1 | 2 | 3 | 4 | 5 | 6 | 7 | 8 |
| S1 Family | S1\_A | - | - | - | - | - | - | - | 18 |
| | S1\_B | 62 | - | - | - | - | - | - | - |
| | S1\_C | - | - | - | - | 12 | - | - | - |
| | S1\_D | - | - | - | - | - | - | - | 12 |
| | S1\_E | - | - | - | - | - | - | - | 16 |
| | S1\_F | - | - | - | - | - | - | - | 27 |
| | S1\_G | - | - | - | - | - | - | - | 8 |
| | S1\_H | 13 | - | - | - | 14 | - | - | 2 |
| | S1\_I | - | - | - | - | - | - | - | 23 |
| | S1\_J | - | - | 105 | - | - | - | - | - |
| | S1\_K | - | - | 1 | - | 61 | - | - | - |
| | S1\_L | 1 | 287 | - | - | - | - | - | - |
| | S1\_M | - | - | - | 178 | - | - | - | - |
| | S1\_N | - | - | - | 7 | - | 94 | - | - |
| | S1\_O | 1 | - | - | - | - | - | 77 | - |
C
G
6
5
4
3
2
1
