# Supplementary material for: Linking anthocyanin diversity, hue, and genetics in purple corn
Source: G3 (Bethesda). 2021 Jan 11;11(2):jkaa062. doi: 10.1093/g3journal/jkaa062 (PMC8022952; doi:10.1093/g3journal/jkaa062)
Supplement: jkaa062_Supplementary_Data [file jkaa062_supplementary_data.zip › Supplementary Figure S6.pptx]

## Slide 1
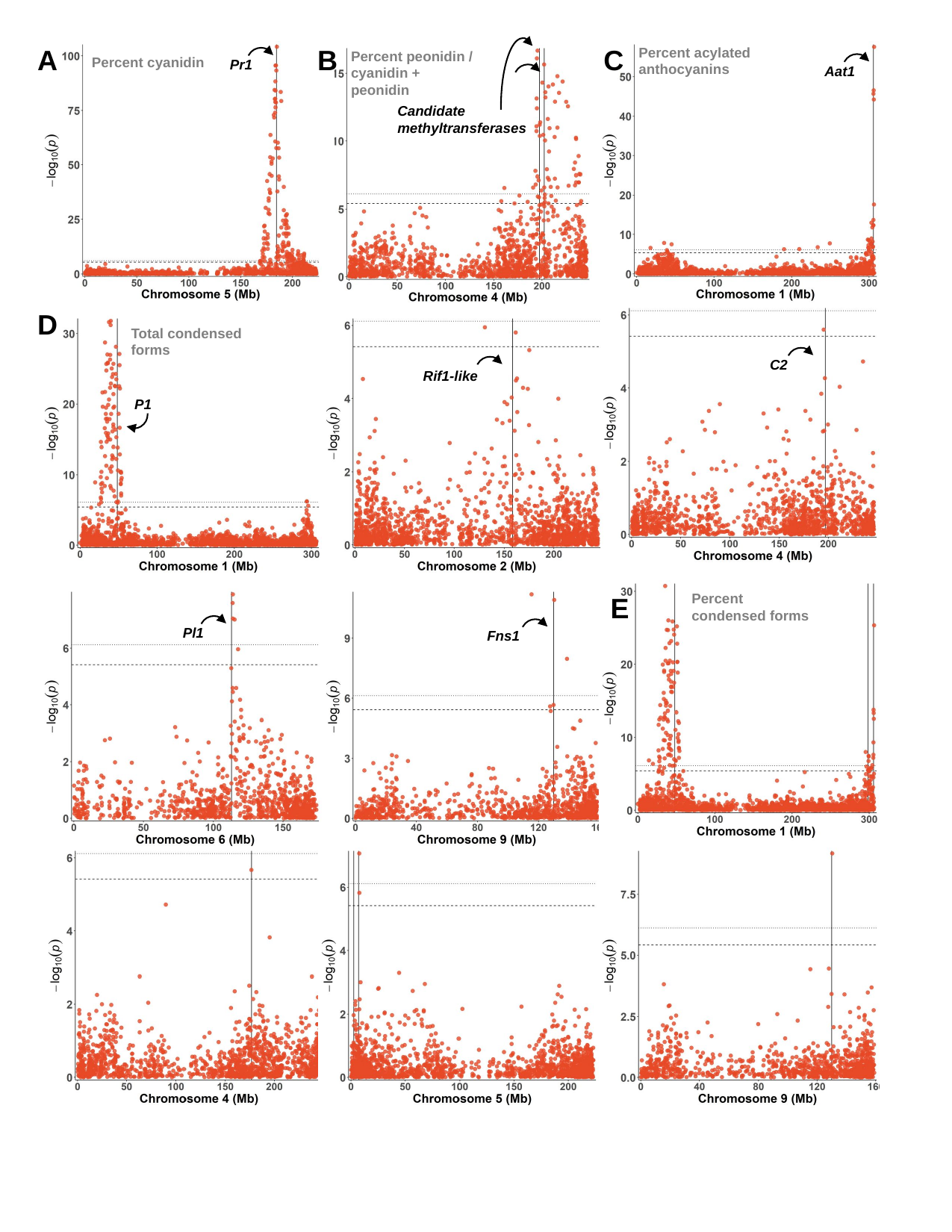

A
B
C
Percent peonidin / cyanidin + peonidin
Candidate methyltransferases
Percent acylated anthocyanins
Aat1
Percent cyanidin
Pr1
D
Total condensed forms
C2
Rif1-like
P1
Percent condensed forms
E
Pl1
Fns1
