# Supplementary material for: Linking anthocyanin diversity, hue, and genetics in purple corn
Source: G3 (Bethesda). 2021 Jan 11;11(2):jkaa062. doi: 10.1093/g3journal/jkaa062 (PMC8022952; doi:10.1093/g3journal/jkaa062)
Supplement: jkaa062_Supplementary_Data [file jkaa062_supplementary_data.zip › Supplementary Figure S8.pptx]

## Slide 1
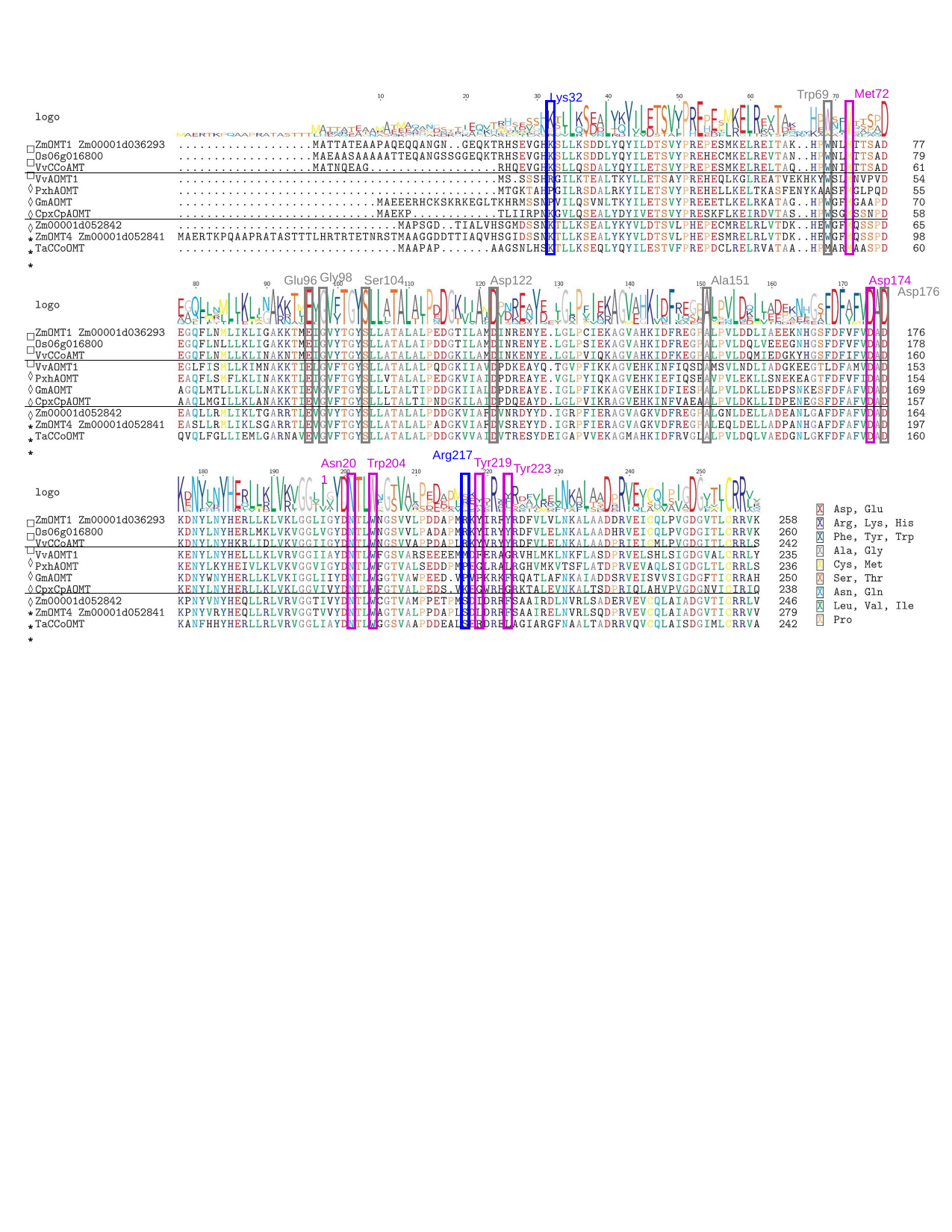

Met72
Trp69
Lys32
Gly98
Glu96
Ser104
Asp122
Ala151
Asp174
Asp176
Arg217
Tyr219
Asn201
Trp204
Tyr223
□□□
◊
◊
◊
◊
*
*
*
□□□
◊
◊
◊
◊
*
*
*
□□□
◊
◊
◊
◊
*
*
*
Y212
Replaced in AOMTs b/c bulky
Y208
Replaced in AOMT b/c bulky
