# Supplementary material for: Linking anthocyanin diversity, hue, and genetics in purple corn
Source: G3 (Bethesda). 2021 Jan 11;11(2):jkaa062. doi: 10.1093/g3journal/jkaa062 (PMC8022952; doi:10.1093/g3journal/jkaa062)
Supplement: jkaa062_Supplementary_Data [file jkaa062_supplementary_data.zip › Supplementary Figure S9.pptx]

## Slide 1
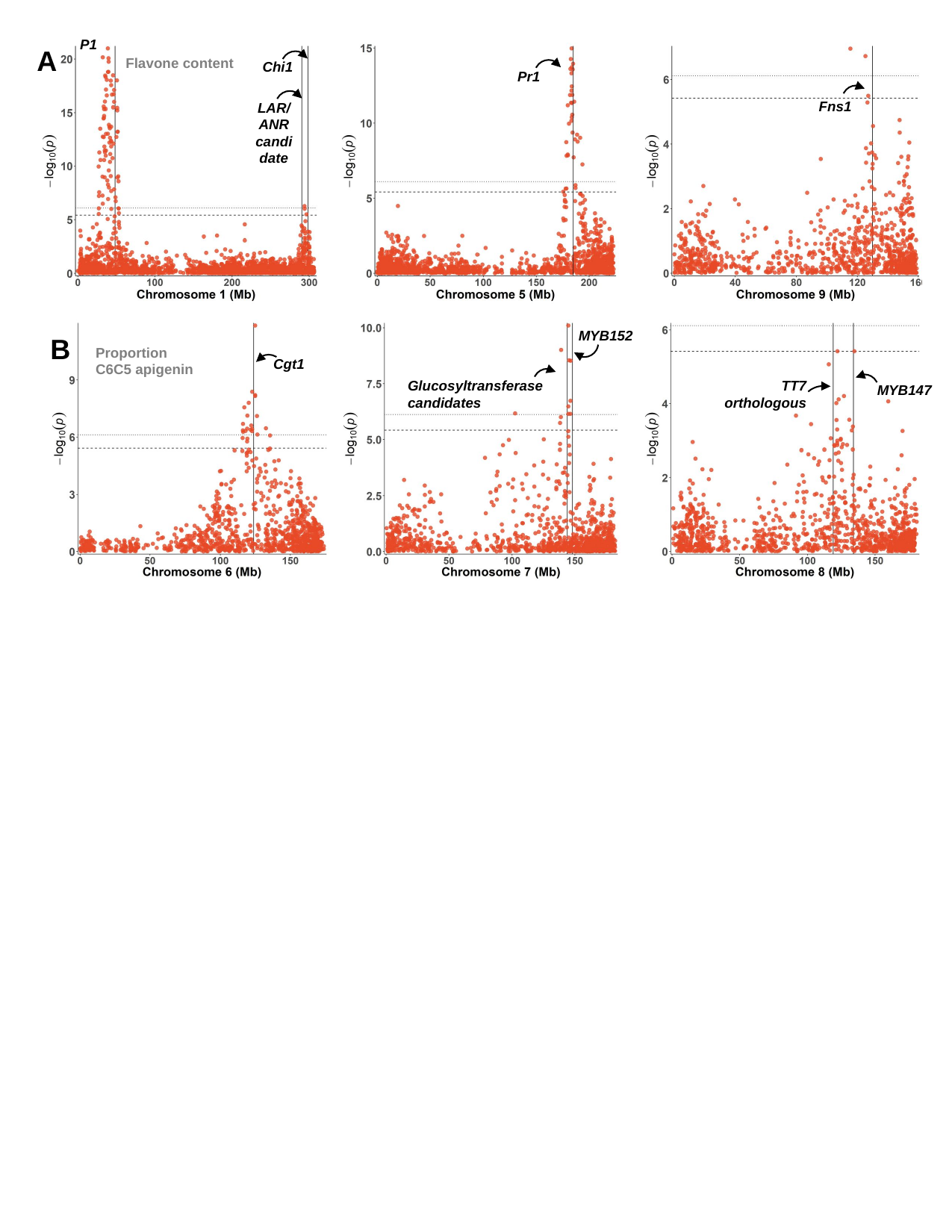

P1
A
Flavone content
Chi1
Pr1
Fns1
LAR/ANR candidate
MYB152
B
Proportion C6C5 apigenin
Cgt1
Glucosyltransferase candidates
TT7 orthologous
MYB147
