# Supplementary material for: Linking anthocyanin diversity, hue, and genetics in purple corn
Source: G3 (Bethesda). 2021 Jan 11;11(2):jkaa062. doi: 10.1093/g3journal/jkaa062 (PMC8022952; doi:10.1093/g3journal/jkaa062)
Supplement: jkaa062_Supplementary_Data [file jkaa062_supplementary_data.zip › Supplementary Table S1.docx]

**Supplementary Table 1:** Description of proteins used for methyltransferase phylogram (Supplementary Figure 7) and alignment (Supplementary Figure 8).

| **Species** | **Identifier** | | **Genbank ID / MaizeGDB ID** | **Gene product action** |
| --- | --- | --- | --- | --- |
| *Arabidopsis thaliana* | At1g67980 | | NP_564916.2 | Caffeoyl-CoA 3-O-methyltransferase |
| *Arabidopsis thaliana* | At4g26220 | | AAM64800.1 | Caffeoyl-CoA O-methyltransferase-like |
| *Arabidopsis thaliana* | At4g34050 | | NP_195131.1 | Caffeoyl coenzyme A O-methyltransferase |
| *Chrysoplenium americanum* | CaFOMT | | AAA80579.1 | 3' flavonoid O-methyltransferase |
| *Cyclamen persicum x Cyclamen purpurascens* | CkmOMT2 | | BAK74804.1 | Anthocyanin O-methyltransferase |
| *Cyclamen persicum x Cyclamen purpurascens* | CkmOMT1 | | BAK74803.1 | Caffeoyl CoA O-methyltransferase-like |
| *Dorcoceras hygrometricum* | DhAOMT | | KZV17033.1 | Anthocyanin O-methyltransferase |
| *Glycine max* | GmAOMT | | NP_001242455.2 | Anthocyanin 3'-O-methyltransferase |
| *Medicago sativa* | MsCCoAOMT | | Q40313.1 | trans-caffeoyl-CoA 3-O-methyltransferase |
| *Medicago sativa* | MsCAOMT | | AAB46623.1 | Caffeic acid 3-0-methyltransferase |
| *Mesembryanthemum crystallinum* | McPFOMT | | AAN61072.1 | O-methyltransferase (Caffeoyl CoA, Flavonols, Flavones, Caffeic acid ) |
| *Nemophila menziesii* | NmAMT | | BBA68558.1 | anthocyanin methyltransferase |
| *Nicotiana tabacum* | NtCcoAOMT | | AAC49913.1 | Caffeoyl-coenzymeA O-methyltransferase |
| *Nicotiana tabacum* | NtCCoAOMT5 | | AAB80931.1 | Caffeoyl-CoA 3-O-methyltransferase |
| *Nicotiana tabacum* | NtCAOMT | | AAL91506.1 | Caffeic acid O-methyltransferase II |
| *Oryza sativa* | Os06g016800 | | NP_001056910.1 | Caffeoyl-CoA 3-O-methyltransferase (similar to) |
| *Oryza sativa* | OsROMT | | ABB90678.1 | Flavonoid 3'-O-methyltransferase |
| *Petroselinum crispum* | PcCoAMT | | AAA33851.1 | Caffeoyl-CoA 3-O-methyltransferase |
| *Petunia integrifolia subsp inflata* | PiAMT | | AIE77050.1 | Anthocyanin methyltransferase |
| *Petunia x hybrida* | PxhCCoMT | | ALP75648.1 | Caffeoyl CoA O-methyltransferase |
| *Petunia x hybrida* | PxhAMT | | AIE77047.1 | Anthocyanin methyltransferase |
| *Populus trichocarpa* | PtCCoAOMT | | CAA12200.1 | Caffeoyl-CoA 3-O-methyltransferase |
| *Populus trichocarpa* | PtCCoAOMT1 | | ACC63876.1 | trans-Caffeoyl- CoA 3-O-methyltransferase |
| *Solanum lycopersicum* | SlAOMT | | AIS23587.1 | Anthocyanin O-methyltransferase |
| *Solanum tuberosum* | StAOMT | | AGC31679.1 | Anthocyanin O-methyltransferase |
| *Solanum tuberosum* | StCCoAOMT | | BAC23054.1 | Caffeoyl-CoA O-methyltransferase |
| *Stellaria longipes* | SlCCoAMT | | AAB61680.1 | Caffeoyl CoA, Flavonol, Flavone, Caffeic esters |
| *Triticum aestivum* | TaCCoOMT | | AXM42901.1 | Caffeoyl CoA O-methyltransferase |
| *Vinis vinifera* | VvCCoAMT | | CAA90969.1 | Caffeoyl CoA O-methyltransferase |
| *Vitis vinifera* | VvAOMT1 | | ACO52469.1 | Anthocyanin O-methyltransferase |
| *Vitis vinifera* | VvAOMT3 | | CBI27641.3 | Anthocyanin O-methyltransferase |
| *Vitis vinifera* | VvCAOMT | | AAF44672.1 | Caffeic acid O-methyltransferase |
| *Zea mays* |  | | Zm00001d052683 |  |
| **Supp. Table 1 (cont.)** | |  |  |  |
| *Zea mays* |  | | Zm00001d052684 |  |
| *Zea mays* |  | | Zm00001d052842 |  |
| *Zea mays* |  | | Zm00001d052843 |  |
| *Zea mays* | ZmOMT1 | | Zm00001d036293 | Caffeoyl CoA O-methyltransferase |
| *Zea mays* | ZmOMT2 | | Zm00001d045206 | Caffeoyl CoA O-methyltransferase |
| *Zea mays* | ZmOMT3 | | Zm00001d024596 | Caffeoyl CoA O-methyltransferase |
| *Zea mays* | ZmOMT4 | | Zm00001d052841 | Caffeoyl CoA O-methyltransferase |
| *Zinnia violaceae* | ZvCcoAMT | | AAA59389.1 | trans-Caffeoyl- CoA 3-O-methyltransferase |
