# Supplementary figures and images for: Linking anthocyanin diversity, hue, and genetics in purple corn
Source: G3 (Bethesda). 2021 Jan 11;11(2):jkaa062. doi: 10.1093/g3journal/jkaa062 (PMC8022952; doi:10.1093/g3journal/jkaa062)

## Slide 1
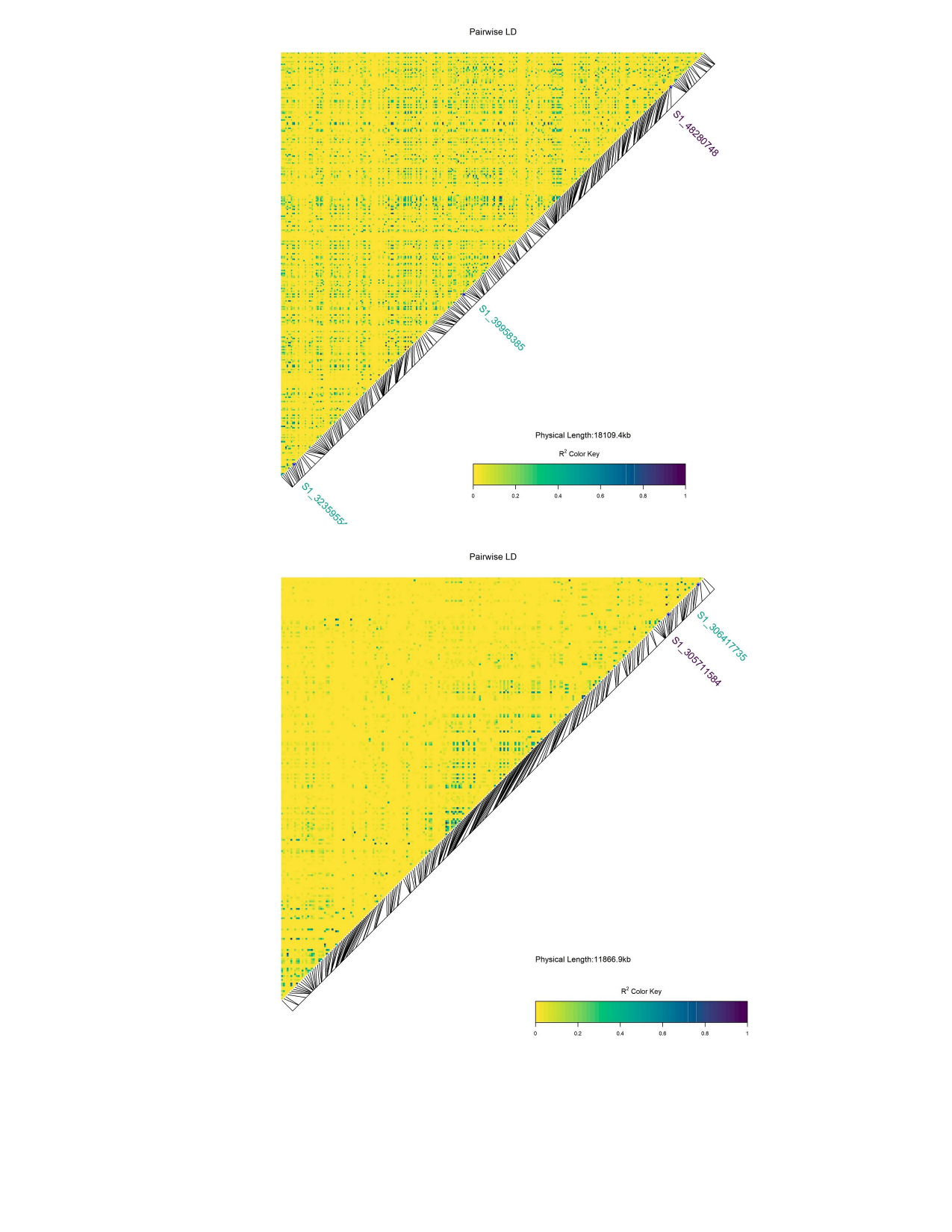

Supplement: jkaa062_Supplementary_Data [file jkaa062_supplementary_data.zip › Supplementary Figure S10.pptx]

## Slide 1
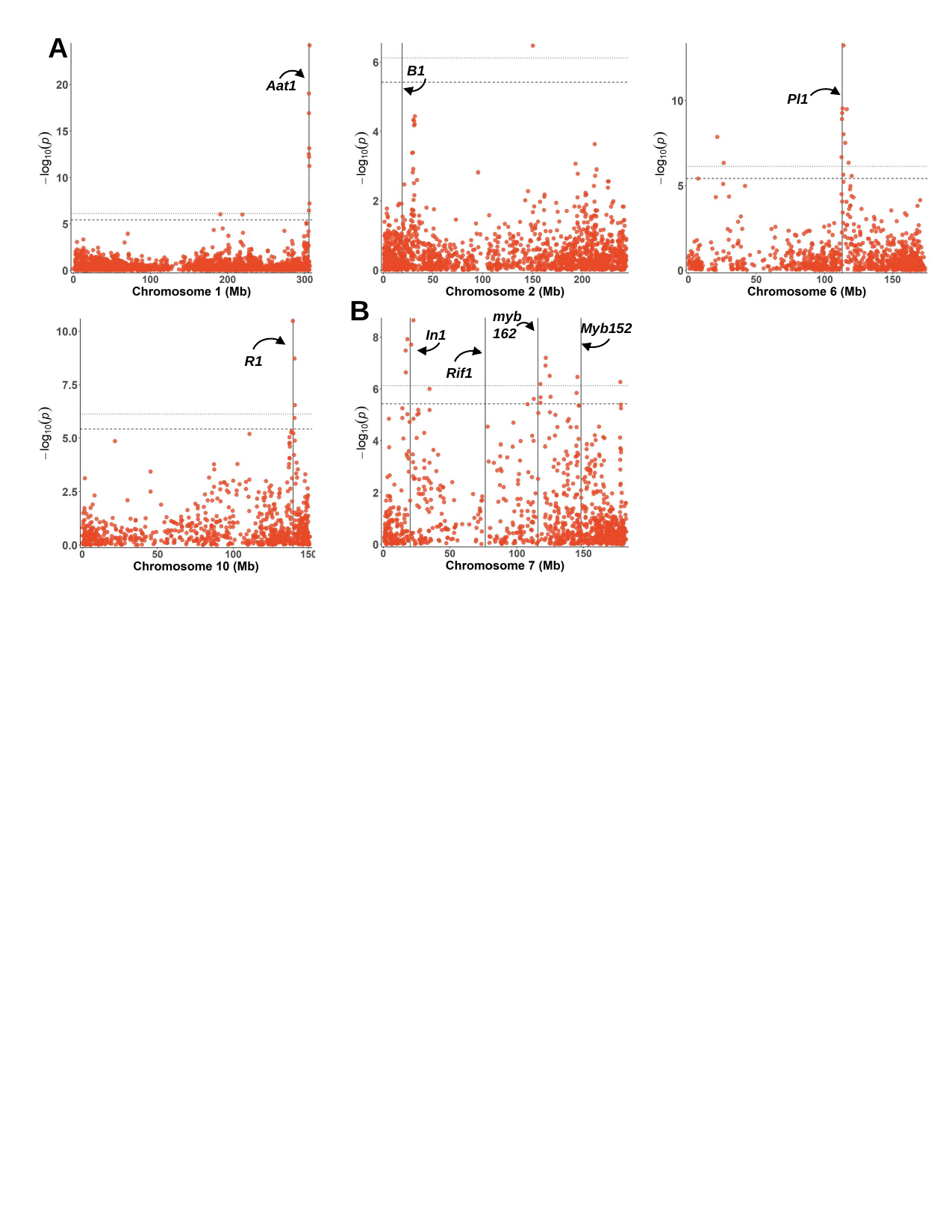

A
Aat1
B1
Pl1
B
myb162
Myb152
In1
R1
Rif1

Supplement: jkaa062_Supplementary_Data [file jkaa062_supplementary_data.zip › Supplementary Figure S11.pptx]

## Slide 1
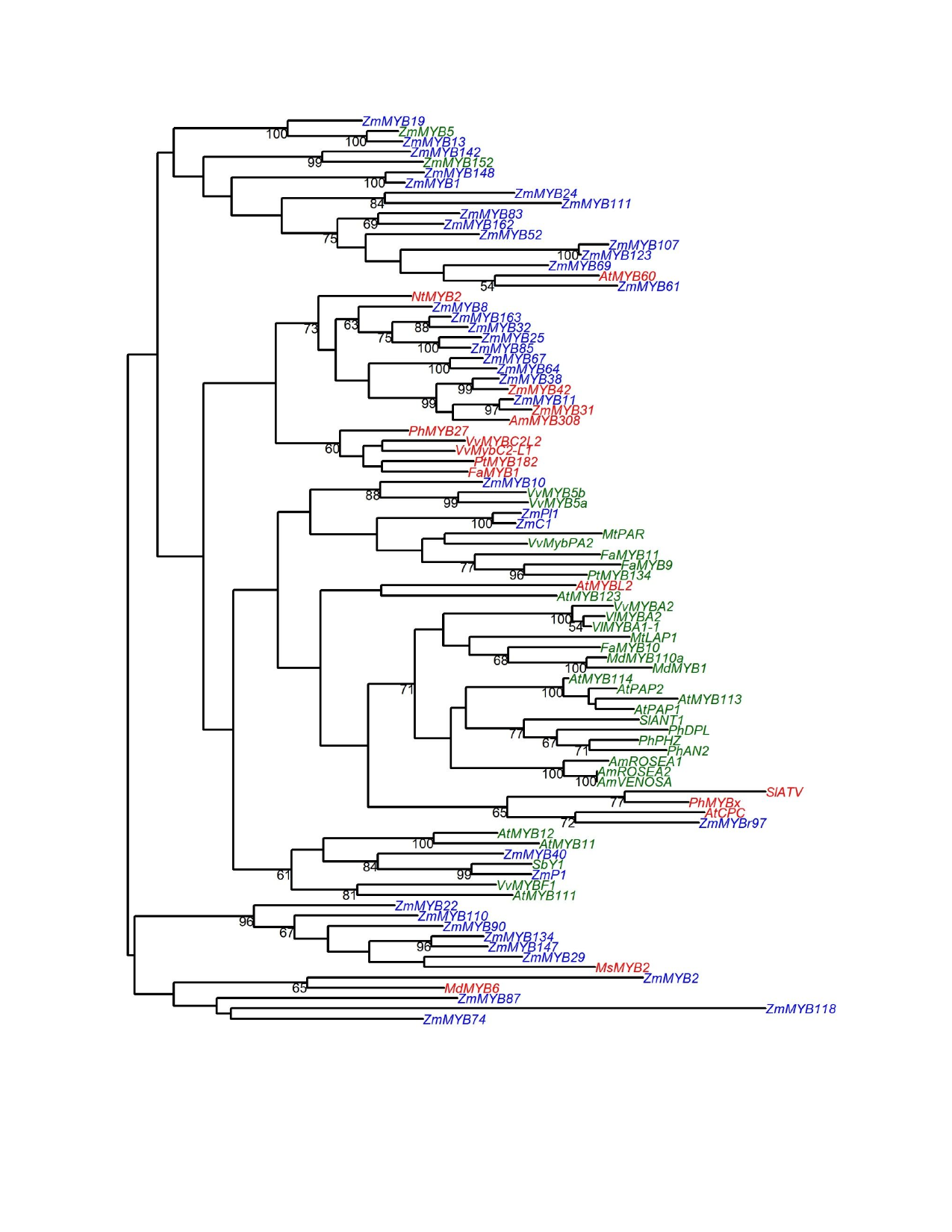

Supplement: jkaa062_Supplementary_Data [file jkaa062_supplementary_data.zip › Supplementary Figure S12.pptx]

## Slide 1
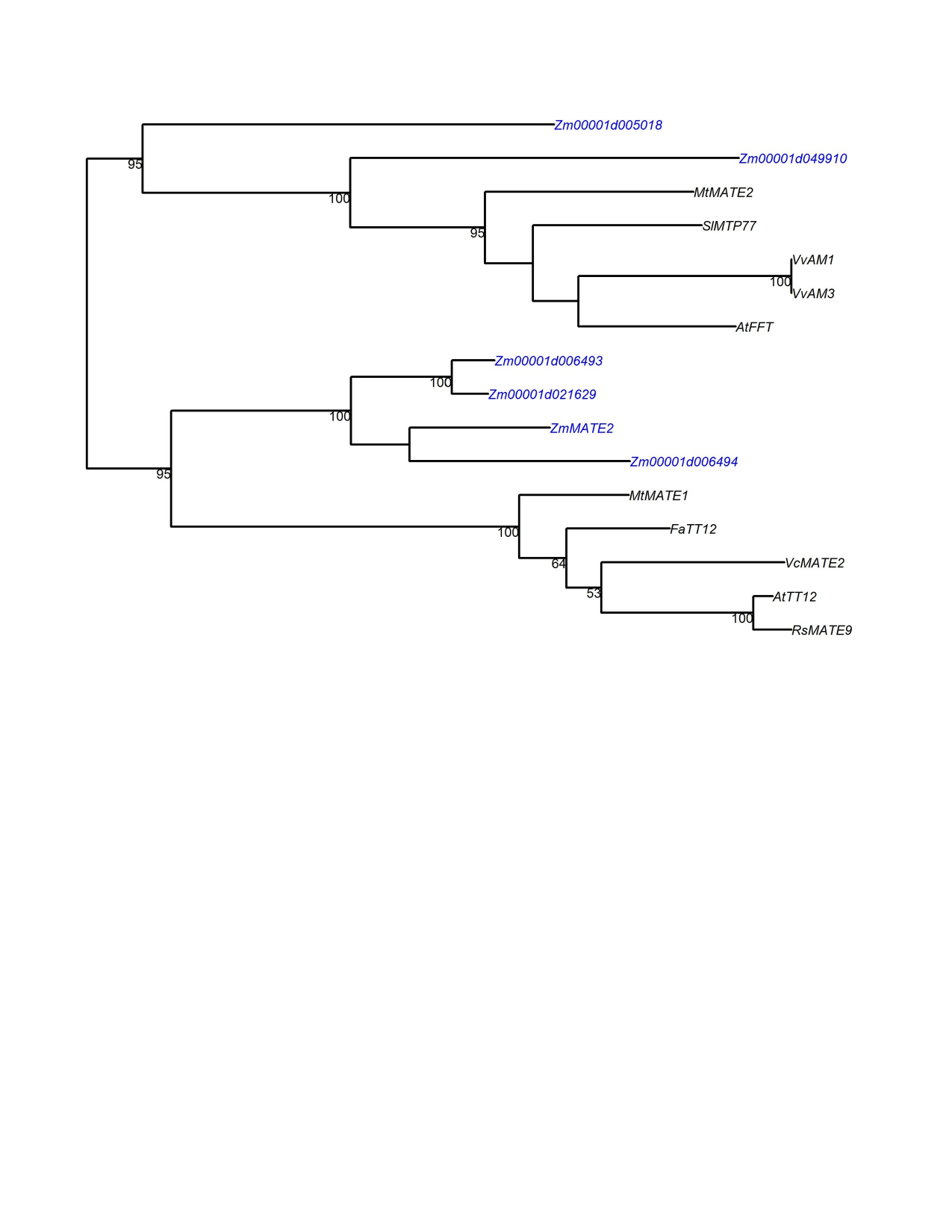

Supplement: jkaa062_Supplementary_Data [file jkaa062_supplementary_data.zip › Supplementary Figure S13.pptx]

## Slide 1
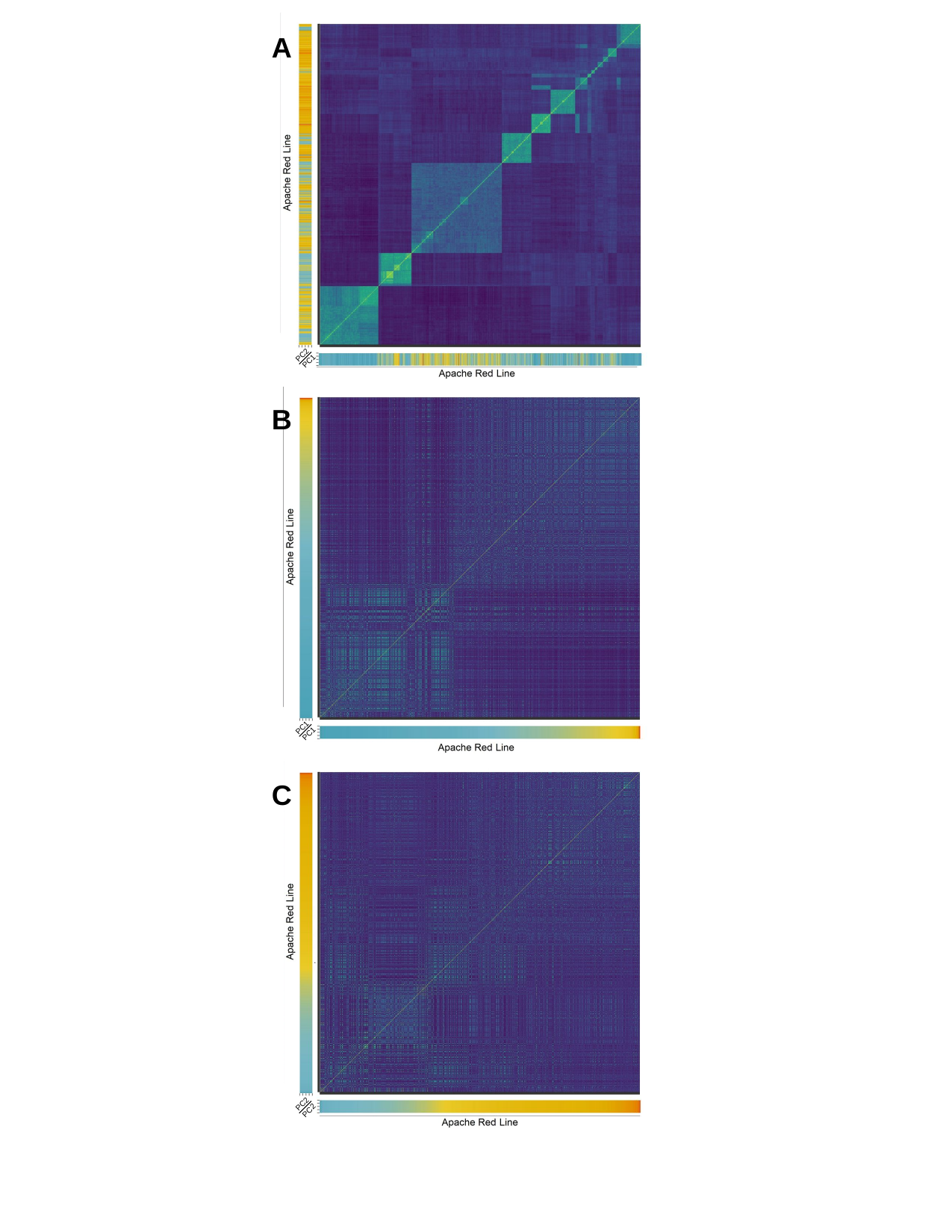

A
PC2
PC1
B
PC1
PC1
C
PC2
PC2

Supplement: jkaa062_Supplementary_Data [file jkaa062_supplementary_data.zip › Supplementary Figure S3.pptx]

## Slide 1
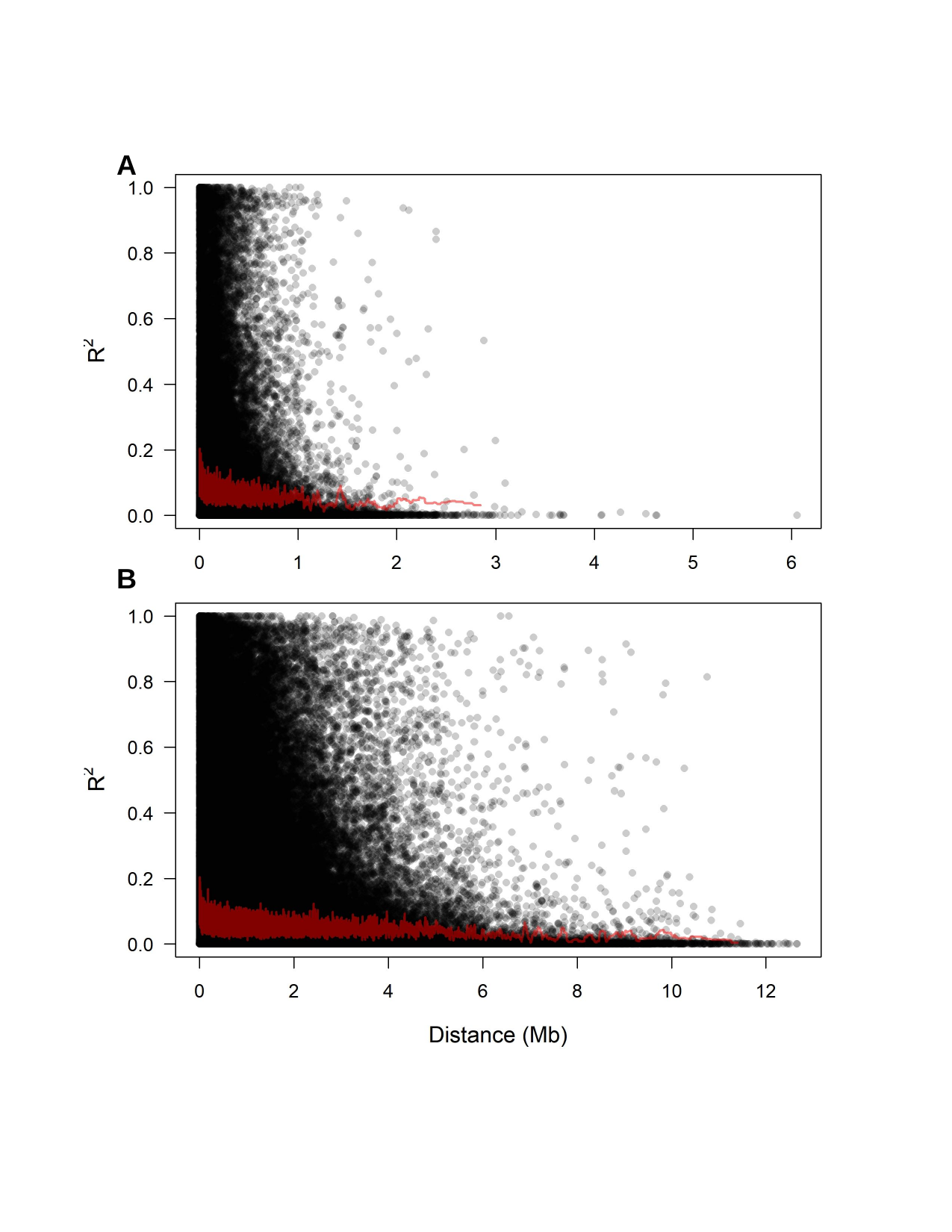

A
B

Supplement: jkaa062_Supplementary_Data [file jkaa062_supplementary_data.zip › Supplementary Figure S5.pptx]

## Slide 1
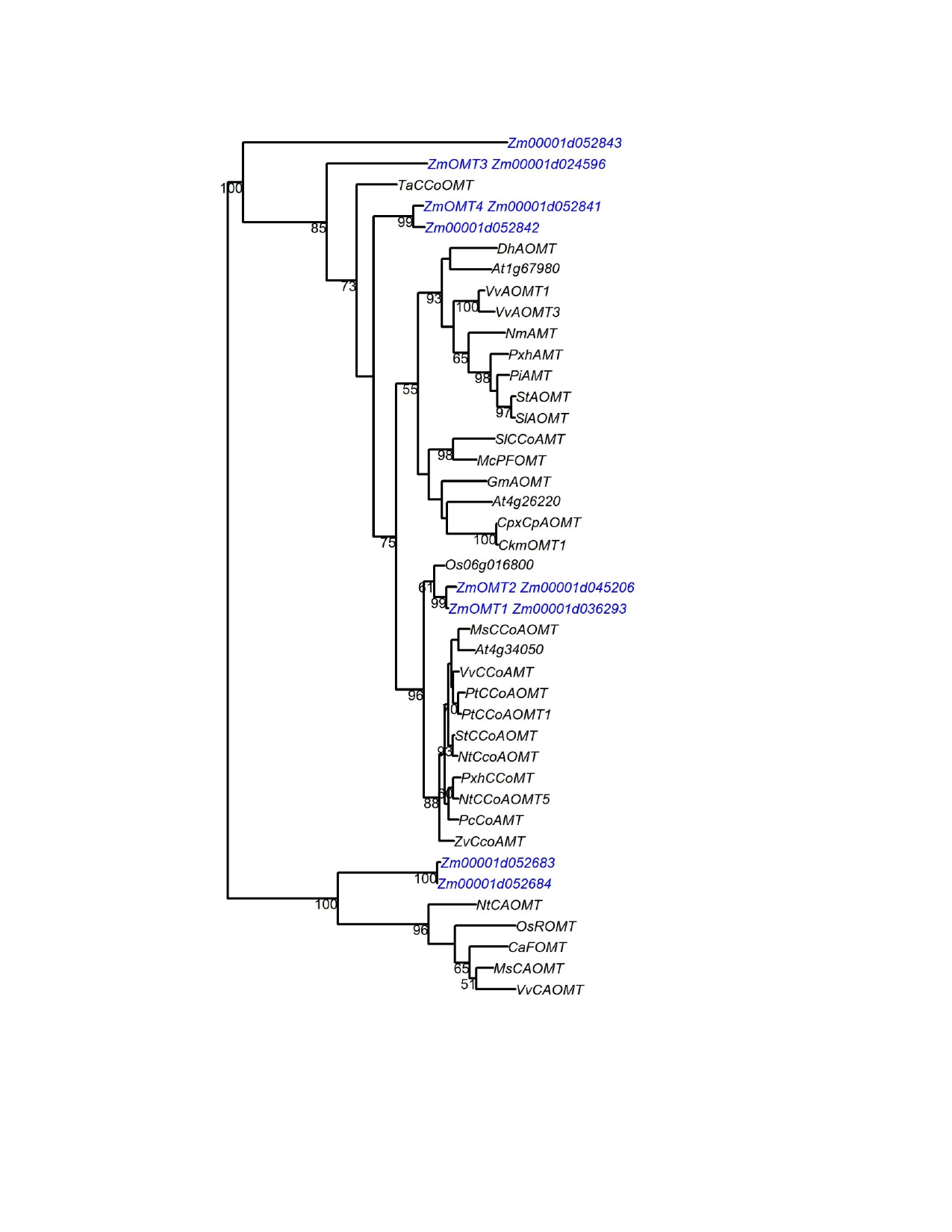

Supplement: jkaa062_Supplementary_Data [file jkaa062_supplementary_data.zip › Supplementary Figure S7.pptx]
